# Optimal vitamin D supplement dosage for improving insulin resistance in children and adolescents with overweight/obesity: a systematic review and network meta-analysis

**DOI:** 10.1007/s00394-023-03301-x

**Published:** 2023-12-30

**Authors:** Linlian Zhu, Shan Li, Lijuan Zhong, Shiping Xu, Hongbin Zhu

**Affiliations:** 1 Department of Nutriology, Dongtai People’s Hospital, Dongtai, 224200 Jiangsu Province China; 2 Department of Integrated TCM and Western Medicine, Dongtai People’s Hospital, Dongtai, 224200 Jiangsu Province China; 3 Department of Endocrinology, Dongtai People’s Hospital, Dongtai, 224200 Jiangsu Province China; 4 Department of Cardiovascular Medicine, Dongtai People’s Hospital, Dongtai, 224200 Jiangsu Province China

**Keywords:** Children, Adolescent, Overweight, Obesity, Vitamin D

## Abstract

**Purpose:**

We conducted a network meta-analysis which aims to evaluate the comparative efficacy of different supplementation dosages of vitamin D on cardiometabolic and bone-metabolic indicators as well as insulin resistance in children and adolescents with overweight/obesity.

**Methods:**

Eligible studies published before December 10, 2022 were retrieved from PubMed, EMBASE, Cochrane Library, and Web of Science. Mean difference and 95% confidence interval (CI) were used to express pooled estimates. Network meta-analysis of multiple doses, including low (< 1000 IU/day, LDS), medium (1000–2000 IU/day, MDS), high (2000–4000 IU/day, HDS), and extremely high (> 4000 IU/day, EHDS) dosage strategy, was conducted using STATA/MP 14.0.

**Results:**

Our network meta-analysis of 15 RCTs suggested that, compared with placebo and LDS, EHDS was increased 25-(OH)-D, with a pooled MD of 8.65 (95% CI 4.72–12.58) and 7.66 (95% CI 0.91–14.41), respectively. Meanwhile, EHDS also decreased ho meostasis model assessment-insulin resistance (HOMA-IR) (MD: − 0.74; 95% CI: − 1.45 to − 0.04) and C-reactive protein (CRP) (MD: − 18.99; 95% CI − 21.60 to − 16.38), and EHDS was also better than LDS (MD: − 18.47; 95% CI − 20.66 to − 16.28) and MDS (MD: − 19.69; 95% CI − 22.17 to − 17.21) in decreasing CRP. Ranking probability suggested that EHDS ranked best for increasing 25-(OH)-D, and decreasing HOMA-IR and CRP, with a probability of 86.1%, 83.1%, and 76.6%, respectively.

**Conclusions:**

The results of our network meta-analysis suggest that EHDS may be the best strategy for vitamin D supplementation to reduce inflammatory responses as well as improve insulin resistance in children and adolescents with overweight/obesity.

**PROSPERO registration number:**

CRD42023387775.

**Supplementary Information:**

The online version contains supplementary material available at 10.1007/s00394-023-03301-x.

## Introduction

Childhood overweight and obesity is a significant global public health issue, which is highly prevalent in developed countries and gains an increasing prevalence rate in low- and middle-income countries [1]. In 2019, it was estimated that 206 million children and teenagers would suffer from obesity in 2025, and the number would hit 254 million in 2030 [2] It has been reported that metabolic syndrome, an increase in insulin resistance, and poor glucose tolerance are linked to childhood obesity [3]. Type-2 diabetes (T2DM), psychosocial comorbidity, and cardiometabolic diseases are of higher possibility to be developed by children and adolescents with insulin resistance and metabolic syndrome than those with normal body mass index (BMI) [4–6].

It is necessary to conduct an early intervention, as there is a high risk of complications among children and adolescents with obesity. However, in most cases, medication administration and surgical interventions are not applicable to children [7]. Therefore, exploring efficient alternative treatments is urgently needed. The relationship between childhood obesity and reduced vitamin D concentration has been widely documented, even though the pathophysiology of children with overweight/obesity and adolescents is complicated and multivariant [8, 9]. Therefore, it arouses researchers’ interest in studying the effect of vitamin D supplementation.

As a fat-soluble steroid pro-hormone with autocrine, paracrine, and endocrine action, Vitamin D can regulate musculoskeletal metabolism, and modulate glucose homeostasis, inflammation response, insulin secretion, and cardiovascular metabolism [10]. So far, numerous studies have shown that vitamin D deficiency increases the risk of metabolic disorders, such as insulin resistance, dyslipidemia, and cardiovascular illnesses in children and adolescents with overweight/obesity [11–13]. Further evidence of the effect of vitamin D supplementation in reducing insulin resistance and enhancing cardio-metabolism in children and adolescents with overweight/obesity comes from a recent pair-wise meta-analysis [14]. In addition, many studies have also demonstrated the safety profile of vitamin D supplementation in different populations, even at high-dose daily vitamin D supplementation [15]. A monthly intake of 300,000 IU of vitamin D3 by hemodialysis patients aged 18 and above for 9 months [16] and a meta-analysis have demonstrated the safety of pediatric patients aged 0–18 years taking 400 IU/d of vitamin D [17]. Nevertheless, the dosage levels of vitamin D supplementation vary in clinical practice, and consensus on their comparative efficacy has not been reported yet, making decision-making very difficult. The traditional meta-analysis cannot compare dosage strategies that have never been compared directly before, nor simultaneously compare more than two dosage levels. Although the recent pair-wise meta-analysis introduced subgroup analysis, the relative effectiveness of various vitamin D supplementation dose regimens still needs to be determined.

Network meta-analysis (NMA) can simultaneously combine direct and indirect comparisons to obtain greater statistical power [18], and can also give a relative ranking of treatments and facilitate the comparisons between collected interventions [19]. Therefore, we aimed to introduce a network meta-analytic technique to determine the relative efficacy of the currently available dosage strategies for vitamin D supplementation on cardiometabolic and bone-metabolic indicators and insulin resistance in children and adolescents with overweight/obesity.

## Materials and methods

### Study design

We performed this network meta-analysis in compliance with the Preferred Reporting Items for Systematic Reviews and Meta-Analyses (PRISMA) for network meta-analysis (PRISMA-NMA) [20]. Institutional ethics approval or participants’ informed consents was not required as all statistical analyses were conducted with published data.

### Selection criteria

We selected eligible studies for data analysis according to the following criteria: (i) children or adolescents were confirmed with overweight/obesity based on the Centers for Diseases Control and Prevention criteria; (ii) participants in the study group received the supplementation with one dose of vitamin D, regardless of form, duration, or route of administration; (iii) participants in the control group received the supplementation with another one dose of vitamin D or placebo; (iv) studies reported at least one of the following outcomes, including serum 25-(OH)-D, BMI, homeostasis model assessment-insulin resistance (HOMA-IR), C-reactive protein (CRP), high-density lipoprotein (HDL), low-density lipoprotein (LDL), triglyceride (TG), total cholesterol (TC), parathyroid hormone (PTH), and serum calcium (Ca); and (v) randomized controlled trials (RCTs).

We excluded studies according to the following criteria: (i) ineligible study designs, such as case reports, case series, conference abstracts, or other observational studies; (ii) studies defined overweight/obesity poorly; (iii) study with the number of participants less than 10; and (iv) duplicate report of the same study.

### Literature retrieval

We systematically searched PubMed, EMBASE, Cochrane library, and Web of Science from their inceptions to December 10, 2022 using the following terms and their analogous: “child,” “adolescent,” “overweight,” “obesity,” “vitamin D,” and “random.” The combination of Medical Subject Headings (MeSH) with text words was used to construct search strategy. The search was limited to English. The details of search strategies for all databases are available in supplementary Table 1. We also manually searched the reference lists of topic-related reviews and eligible studies for identifying additional studies.

### Study selection

We exported all references retrieved from the electronic literature search to EndNote X9. Duplicates were first removed automatically using this software. Two independent authors (Linlian Zhu and Shan Li) then screened titles and abstracts of all studies retained from the previous step to initially assess their eligibility. Finally, these two authors downloaded the full texts of all potentially eligible studies which were kept from the title and abstract screening for further eligibility assessment. The consensus principle was introduced to resolve between-authors discrepancies during the study selection.

### Data extraction

Two independent authors (Linlian Zhu and Shan Li) used a pre-designed standardized data extraction sheet on Microsoft® Excel to collect all essential data. Specifically, the following data were extracted from eligible studies, including study characteristics (first author’s name, year of publication, and country), participant characteristics (sample size, the number of males, mean age, and BMI), outcome measures (25-(OH)-D, BMI, HOMA-IR, CRP, HDL, LDL, TG, TC, PTH, and serum Ca), and methodological quality metrics. Any disagreements were resolved through discussion. If studies reported results as a median, standardized error, or interquartile range (IQR), the required data were calculated using the recognized formulas [21]. In addition, we estimated data from figures using a web-based program [22], namely *WebPlotDigitizer* version 4.6, if studies did not report numerical results.

### Evidence map

We drew network plot to show evidence map of each outcome evaluated in this network meta-analysis. In the network plot, the solid circle represented the treatment, such as one dosage strategy of vitamin D supplementation and control; and the solid line connecting the two treatments represented the direct comparison. The size of the circle was positive proportional to the accumulated number of participants for one treatment, and the width of the solid line was proportional to the number of direct comparisons connecting the two treatments.

### Risk of bias assessment

Two independent authors (Linlian Zhu and Shan Li) used the revised Cochrane risk of bias tool (RoB 2) [23] to assess the risk of bias of included RCTs from the following five domains: (i) bias arising from the randomization; (ii) bias due to deviations from intended interventions; (iii) bias due to missing outcome data; (iv) bias in measurement of the outcomes; and (v) bias in selection of the reported results. According to the assessment criteria, each domain would be judged as “low risk,” “some concerns,” or “high risk.” The overall risk of one study would be judged as “low risk of bias” if all domains were judged as “low risk”, as “some concerns” if at least one domain had “some concerns” but no had “high risk”, and as “high risk of bias” if at least one domain had “high risk” multiple domains had “some concerns”. We yielded graphical presentation of the results of risk of bias assessment using the online ‘robvis’ application [24]. The consensus principle was introduced to resolve between-author discrepancies during the risk of bias assessment.

### Statistical analysis

We used mean difference (MD) with its respective 95% confidence interval (CI) for all outcomes because all were continuous variables. Transitivity across studies was assessed by comparing the distribution of three major characteristics that could act as effect modifiers across the different pair-wise comparisons [25]. Then, after confirming the transitivity assumption, we conducted random effects network meta-analysis using the “*mvmeta*” command [26]. Before performing network meta-analysis, we assessed global and local inconsistency using the “design-by-treatment” model [27] and the node-splitting method [28], respectively. According to the result of the inconsistency assessment, we selected an appropriate model for the network meta-analysis of each outcome. Based on the results of network meta-analyses, we ranked all dosing strategies of vitamin D supplementation based on their respective ranking probabilities, which were calculated using the surface under the cumulative ranking (SUCRA) [29]. In addition, we performed a loop inconsistency examination using the node-splitting method to evaluate the reliability of pooled results [30, 31]. Finally, a comparison-adjusted funnel plot was drawn to assess the publication bias and small-study effects [32]. All statistical analyses in the current network meta-analysis were conducted using STATA/MP 14.0 (StataCorp LP, College Station, Texas, USA).

## Results

### Literature selection

**Fig. 1:**
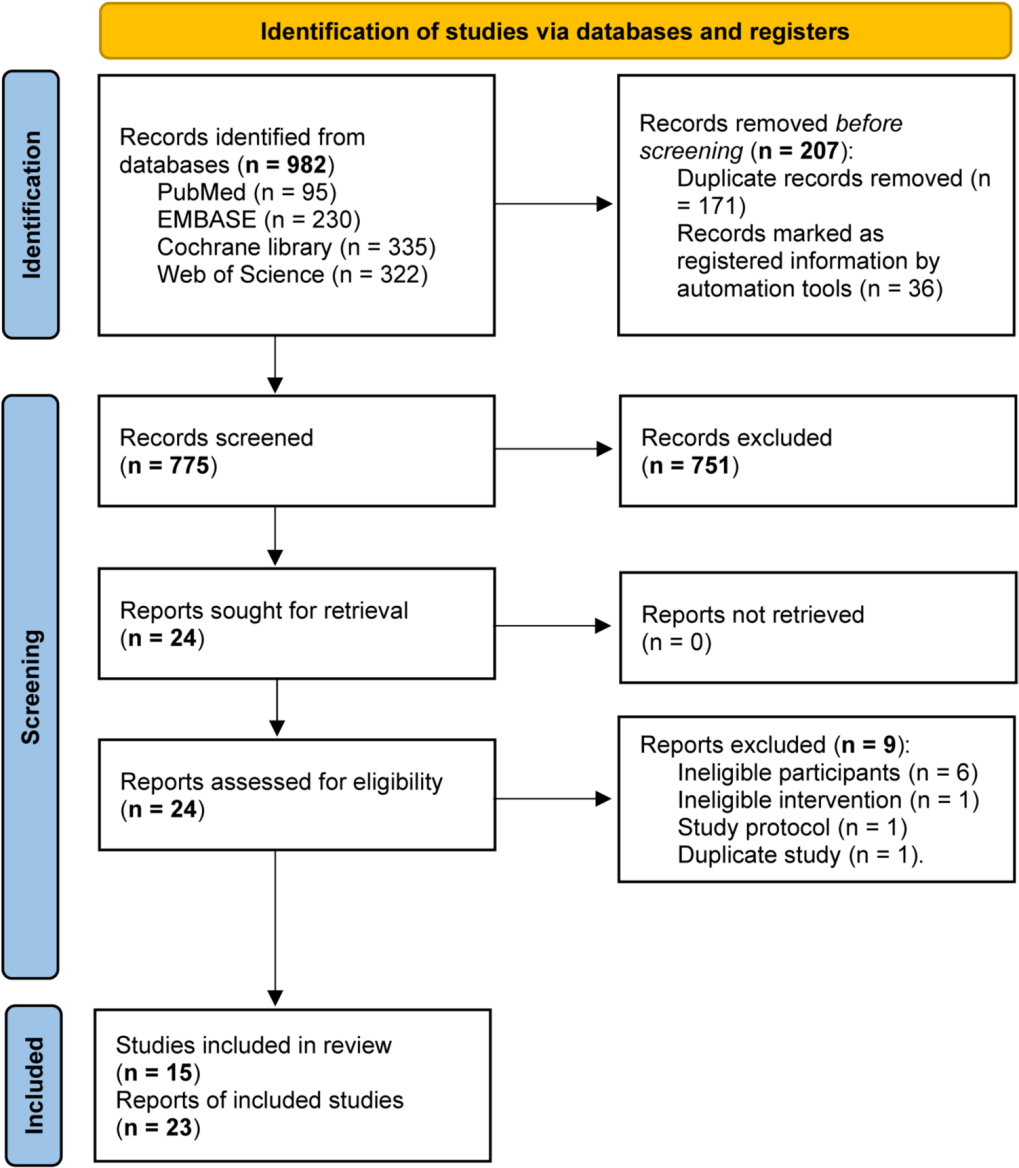
PRISMA flowchart of study retrieval and selection

We identified a total of 982 potentially relevant studies from four target databases. Among these studies, 171 duplicate studies and 36 registered study protocols were first removed automatically by EndNote software. After screening the titles and abstracts of the remaining 775 studies, 24 were kept for further full-text evaluation. After excluding 9 studies due to ineligible participants (*n* = 6), ineligible intervention (*n* = 1), study protocol for an RCT (*n* = 1), and duplicate study (*n* = 1), a total of 15 studies [33–46] were included in this network meta-analysis. The process of study selection is displayed in Fig. 1.

### Study characteristics

All studies we included were published between 2013 and 2022. The number of participants in the individual study ranged from 20 to 378, with a total of 1693 participants. The included studies reported multiple dosage strategies for vitamin D supplementation. We categorized dosage strategy into low (< 1000 IU/day, LDS), medium (1000–2000 IU/day, MDS), high (2000–4000 IU/day, HDS), and extremely high (> 4000 IU/day, EHDS). Based on the classification of dosage strategy, eight studies [34, 35, 37, 38, 40, 44, 45, 47] compared EHDS with control, one study [46] compared LDS with EHDS, one study [43] compared LDS, EHDS and control, three studies [33, 41, 42] compared HDS, MDS and LDS, and two studies [36, 39] compared MDS with control. Other baseline characteristics of all included studies are summarized in Table 1. The outcomes and their respective data of these included studies are summarized in Supplementary Table 2. The evidence plots for 25-(OH)-D, BMI, HOMA-IR, and CRP are shown in Supplementary Fig. 1, and the evidence plots for cardiometabolic factors (HDL, LDL, TG, and TC) and bone metabolism-associated markers (PTH and Ca) are shown in Supplementary Figs. 2 and 3, respectively.

**Table 1:** Baseline characteristics of included studies in this network meta-analysis

Study	Country	Category of intervention	Sample size	Males	Mean age, years	BMI, kg/m2
Vinet et al. 2021 [47]	France	Control	13	5	14	32.7
		EHDS: 4000 IU daily for 3 months	13	5	14.6	33.9
Namakin et al. 2021 [40]	Iran	Control	50	31	n.r	n.r
		EHDS: 50000 IU weekly for 12 weeks	51	23	n.r	n.r
Samaranayake et al. 2020 [43]	Sri Lanka	Control	31	25	10.6	26.6
		LDS: 2500 IU weekly for 24 weeks	33	22	9.8	28.3
		EHDS: 50000 IU weekly for 24 weeks	32	22	9.9	27.2
Brzeziński et al. 2020 [36]	Poland	Control	67	n.r	10.7	24.5
		MDS: 1200 IU daily for 26 weeks	85	n.r	11.1	25
Sethuraman et al. 2018 [44]	US	Control	14	5	15.3	38.2
		EHDS: 50000 IU weekly for 12 weeks	15	2	15.1	35.4
Brar et al. 2018 [35]	US	Control	10	4	15.1	32.7
		EHDS: 300000 IU once	10	4	15.1	3.29
Shah et al. 2015 [45]	US	Control	20	6	13.6	31
		EHDS: 150000 IU twice, once at baseline and another at 12 weeks	20	8	15.1	36
Nader et al. 2014 [39]	US	Control	24	n.r	15	33.9
		MDS: 2000 IU daily for 12 weeks	20	n.r	15.2	35.3
Belenchia et al. 2013 [34]	Columbia	Control	23	11	13.9	38.9
		EHDS: 4000 IU daily for 6 months	21	11	14.6	39.5
Kelishadi et al. 2014 [37]	Iran	Control	22	n.r	n.r	27.8
		EHDS: 300000 IU weekly for 12 weeks	21	n.r	n.r	28.1
Morrissey et al. 2022 [38]	France	Control	13	5	14	32.7
		EHDS: 4000 IU daily for 3 months	13	5	14.6	33.8
Rajakumar et al. 2020 [41]	US	LDS: 600 IU daily for 12 months	76	22	13.5	30.7
		MDS: 1000 IU daily for 12 months	74	29	13.5	30
		HDS: 2000 IU daily for 12 months	75	27	13.9	30.3
Sacheck et al. 2022 [42]	US	LDS: 600 IU daily for 12 months	100	n.r	n.r	n.r
		MDS: 1000 IU daily for 12 months	87	n.r	n.r	n.r
		HDS: 2000 IU daily for 12 months	93	n.r	n.r	n.r
Varshney et al. 2019 [46]	India	LDS: 12000 IU per month for 12 months	96	n.r	13.2	30.4
		EHDS: 120000 IU per month for 12 months	93	n.r	12.9	29.5
Asghari et al. 2021 [33]	Iran	LDS: 600 IU daily for 12 months	120	60	9.2	23.2
		MDS: 1000 IU daily for 12 months	127	66	9.4	23.5
		HDS: 2000 IU daily for 12 months	131	72	9.4	23.3

LDS, low dose strategy; MDS, medium dose strategy; HDS, high dose strategy; EHDS, extremely high dose strategy; BMI, body mass index; n.r., not reported

### Risk of bias assessment

The graphical results of our risk of bias assessment of all included studies are shown in Supplementary Fig. 4. Five studies [39, 40, 44, 45, 47] were judged as “some concerns” because detailed information about random sequence generation was unavailable. All studies were judged as “low risk” in domains 2 and 5. Four studies [33, 36, 41, 42] had “high risk” in domain 3 because they missed participants during treatment. Five studies [35, 37, 44, 45, 47] were judged as “some concerns” because it was unclear whether outcome assessors were blinded. Overall, nine [33, 36, 39–42, 44, 45, 47] of the 15 included studies had a high or unclear risk of bias in one or more domains.

### Transitivity assessment

We assessed the assumption of transitivity by comparing the across-dosage-strategy distribution of supplementation duration, mean age, and BMI. As described in Supplementary Table 3, the transitivity assessment confirmed that three characteristics were evenly distributed across dosage strategies, supporting the assumption of transitivity (*P* > 0.05).

### Meta-analysis of 25-(OH)-D

**Fig. 2:**
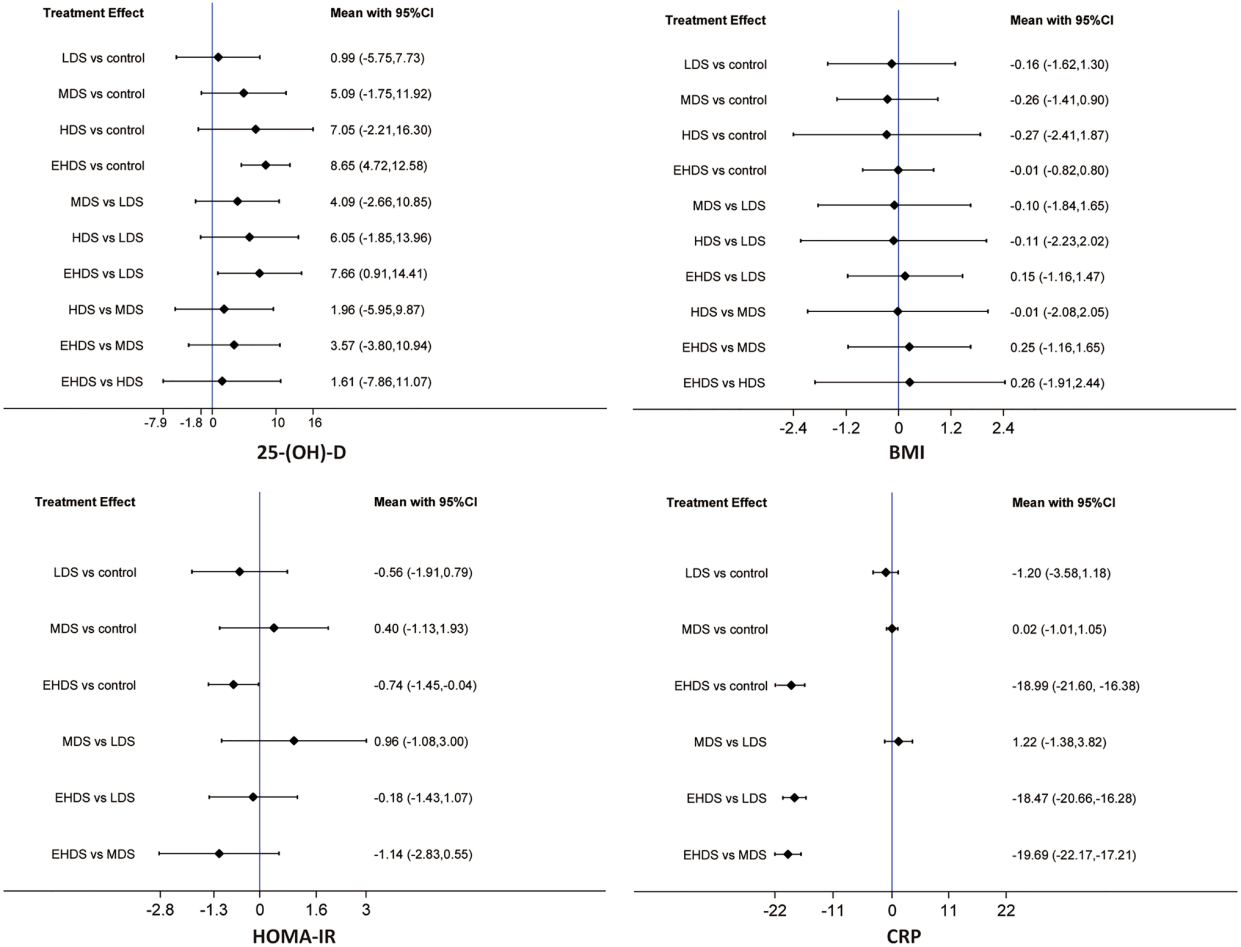
Network meta-analysis of different dosage strategies for vitamin D supplementation in terms of 25-(OH)-D, BMI, HOMA-IR, and CRP. *BMI* body mass index, *HOMA-IR* homeostasis model assessment-insulin resistance, *CRP* C-reactive protein

We selected the consistency model for the network meta-analysis of 25-(OH)-D because global (as shown in Supplementary Fig. 5) and local consistencies (as shown in Supplementary Table 4) were not confirmed. As shown in Fig. 2, pooled results showed that EHDS significantly increased serum concentration of 25-(OH)-D (MD: 8.65; 95% CI 4.72–12.58) and were better than LDS (MD: 7.66; 95% CI 0.91–14.41). All other comparisons between different dosage strategies did not reach statistical significance.

### Meta-analysis of BMI

We performed a network meta-analysis of BMI based on the consistency model because global (as shown in Supplementary Fig. 5) and local inconsistencies (as shown in Supplementary Table 4) were not detected. As shown in Fig. 2, pooled results revealed that all available dosage strategies for vitamin D supplementation did not significantly change BMI and also showed no significant difference between all dosage strategies.

### Network meta-analysis of HOMA-IR

Our network meta-analysis of HOMA-IR was performed based on a consistency model, as the global (as shown in Supplementary Fig. 5) and local consistencies (as shown in Supplementary Table 4) were not identified. As shown in Fig. 2, pooled results revealed that EHDS significantly decreased HOMA-IR (MD: − 0.74; 95% CI: − 1.45 to − 0.04); however, other comparisons did not reach statistical significance.

### Network meta-analysis of CRP

Our network meta-analysis of CRPs was conducted based on inconsistency modeling, as we found significant global (as shown in Supplementary Fig. 5) and local consistencies (as shown in Supplementary Table 4). As shown in Fig. 2, pooled results revealed that EHDS significantly decreased CRP (MD: − 18.99; 95% CI − 21.60 to − 16.38) and were better than LDS (MD: − 18.47; 95% CI − 20.66 to − 16.28) and MDS (MD: − 19.69; 95% CI − 22.17 to − 17.21). However, there was no significant difference for the remaining comparisons.

### Network meta-analysis of HDL

**Fig. 3:**
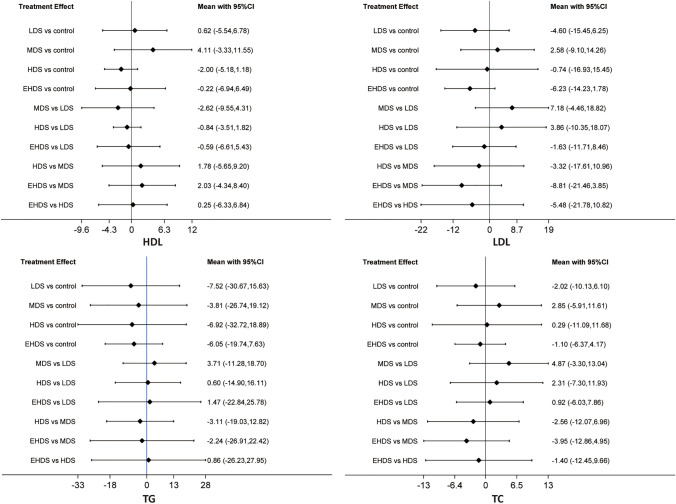
Network meta-analysis of different dosage strategies for vitamin D supplementation in terms of cardiometabolic factors, including HDL, LDL, TG, and TC. *HDL* high-density lipoprotein, *LDL* low-density lipoprotein, *TG* triglyceride, *TC* total cholesterol

We performed the network meta-analysis of HDL based on the inconsistency model because there were significant global (as shown in Supplementary Fig. 6) and local consistencies (as shown in Supplementary Table 4). As shown in Fig. 3, pooled results suggested that all available dosage strategies did not significantly change HDL and also showed no significant difference between all dosage strategies.

### Network meta-analysis of LDL

We performed a network meta-analysis of LDL based on a consistency model, as there was no significant global consistency (as shown in Supplementary Fig. 6) and local consistencies (as shown in Supplementary Table 4). As shown in Fig. 3, our pooled results indicated that all available dosage strategies did not significantly change LDL and also showed no significant difference between all dosage strategies.

### Network meta-analysis of TG

We conducted a network meta-analysis of TG based on a consistency model, as there were no significant global (as shown in Supplementary Fig. 6) and local consistencies (as shown in Supplementary Table 4). As shown in Fig. 3, our pooled results revealed that all available dosage strategies did not significantly change TG and also showed no significant difference between all dosage strategies.

### Network meta-analysis of TC

We conducted a network meta-analysis of TCs based on a consistency model, as there were no significant global (as shown in Supplementary Fig. 6) and local consistencies (as shown in Supplementary Table 4). As shown in Fig. 3, pooled results reported that all available dosage strategies did not significantly change TC and also showed no significant difference between all dosage strategies.

### Network meta-analysis of PTH

**Fig. 4:**
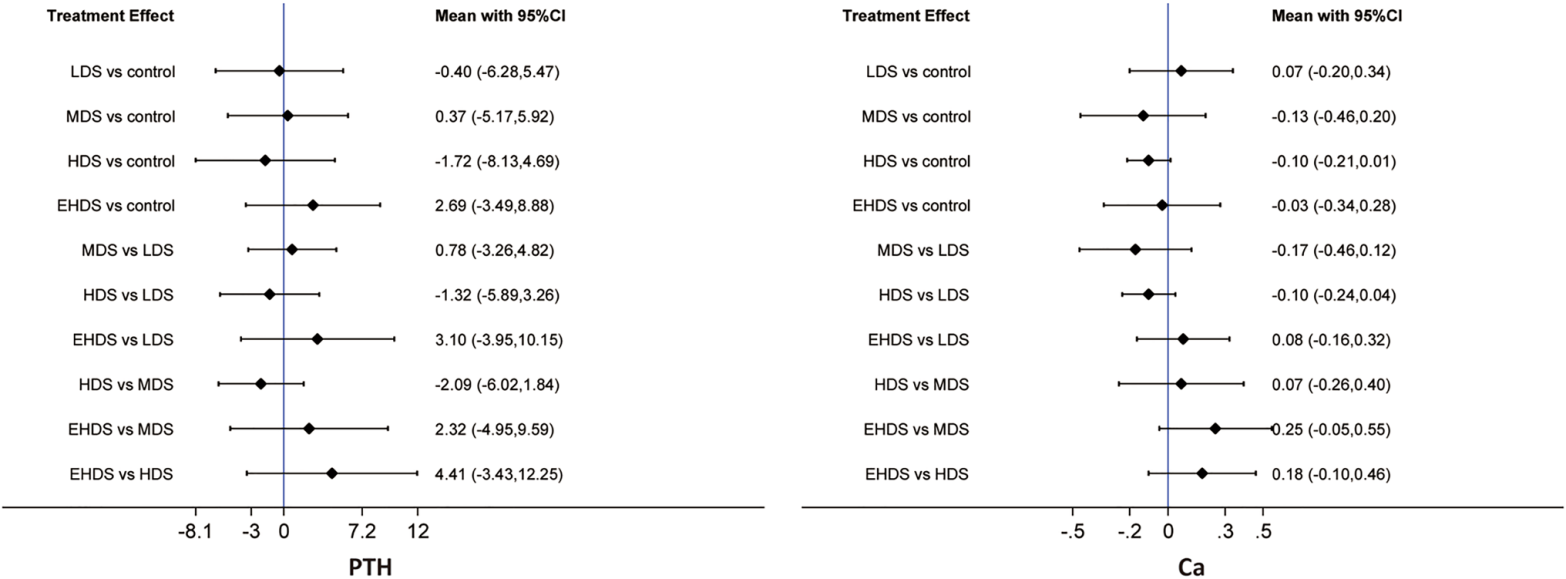
Network meta-analysis of different dosage strategies for vitamin D supplementation in terms of bone metabolism-associated markers, including PTH and Ca. *PTH* parathyroid hormone, *Ca* calcium

We performed a network meta-analysis of PTH based on a coherent model, as there was no significant global (as shown in Supplementary Fig. 7) and local consistencies (as shown in Supplementary Table 4). As shown in Fig. 4, pooled results displayed that all available dosage strategies did not significantly change PTH and also showed no significant difference between all dosage strategies.

### Network meta-analysis of serum Ca

We performed a network meta-analysis of serum calcium based on an inconsistency model, as there were significant global (as shown in Supplementary Fig. 7) and local consistencies (as shown in Supplementary Table 4). As shown in Fig. 4, pooled results revealed that all available dosage strategies did not significantly change serum Ca and also showed no significant difference between all dosage strategies.

### The ranking probability of SUCRA

**Fig. 5:**
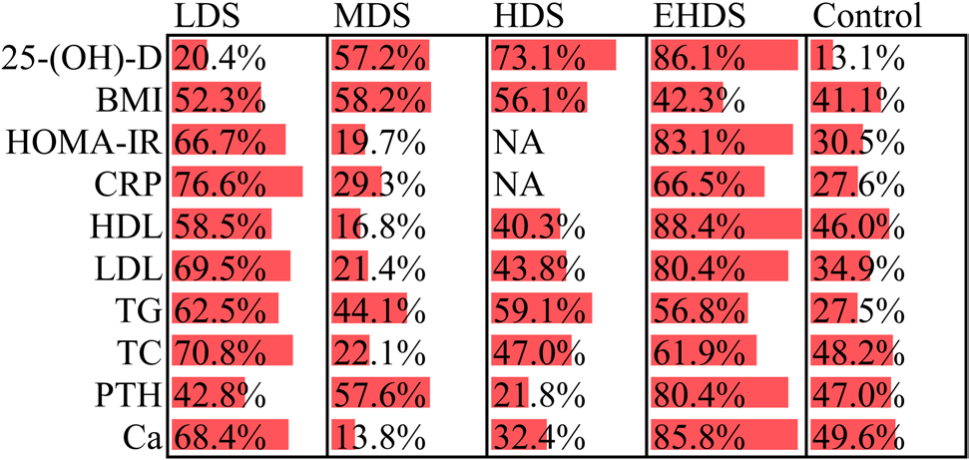
SUCRA results of different dosage strategies in terms of all outcomes. *LDS* low dose strategy, *MDS* medium dose strategy, *HDS* high dose strategy, *EHDS* extremely high dose strategy, *BMI* body mass index, *HOMA-IR* homeostasis model assessment-insulin resistance, *CRP* C-reactive protein, *HDL* high-density lipoprotein, *LDL* low-density lipoprotein, *TG* triglyceride, *TC* total cholesterol, *PTH* parathyroid hormone, *Ca* calcium, *N/A* not applicable

We estimated the ranking probabilities of all available dosage strategies for vitamin D supplementation based on the currently available data. As shown in Fig. 5, from the perspective of numerical results, EHDS ranked first for increasing serum 25-(OH)-D (86.1%), PTH (80.4%), Ca (85.8%) and LDL (80.4%), and for decreasing HOMA-IR (83.1%), CRP (76.6%), and HDL (88.4%), and LDS ranked first for decreasing TG (62.5%) and TC (70.8%). Vitamin D supplementation did not significantly change BMI and also showed no significant difference between all dosage strategies.

#### Safety of intervention

We found that a total of six included studies were analyzed for safety, two of which had a maximum study duration of 12 months [46, 48] and 3 studies [34, 41, 45] observation periods are 6 months, while the shortest is 3 months in one study [44]. Among them, 3 studies [34, 44, 46] administered an ultrahigh dosage exceeding 4000 IU/day for a duration of 3, 6, and 12 months, respectively. Vitamin D supplementation in these studies was found to be safe. None of the study subjects developed noteworthy adverse reactions including symptomatic hypercalcemia and hypercalciuria in these studies.

#### Loop inconsistency

As shown in Supplementary Table 5, we have detected inconsistency for the loop consisting of control-LDS-MDS in the network meta-analysis of serum 25-(OH)-D and the loop consisting of control-LDS-EHDS in the network meta-analysis of CRP, suggesting that pooled results of these two outcomes should be interpreted with more cautions. However, for the network meta-analyses of the remaining outcomes, inconsistency was not detected for all available closed loops, so pooled results were more reliable.

#### Publication bias

We performed network meta-analysis of serum 25-(OH)-D, BMI, HOMA-IR, and CRP, as shown in Supplementary Fig. 8, all comparison-adjusted funnel plots were asymmetric, revealing that there was publication bias for these outcomes. Similarly, as shown in Supplementary Fig. 9, all comparison-adjusted funnel plots for cardiometabolic factors were also asymmetric, suggesting that publication bias might compromise the reliability of these results. However, as shown in Supplementary Fig. 10, all comparison-adjusted funnel plots were symmetric for bone metabolism-associated markers, so pooled results of these outcomes might not be negatively affected by publication bias.

## Discussion

Our study is the first to simultaneously compare currently available dosage strategies for vitamin D supplementation in children and adolescents with overweight or obesity. We included 15 eligible RCTs for the network meta-analysis, involving 1693 children and adolescents with overweight/obesity. Our main finding was that, compared with placebo and LDS, EHDS significantly increases the serum concentration of 25-(OH)-D. In addition, our results also suggest that EHDS significantly decreases HOMA-IR and CRP, and are also superior to LDS and MDS for decreasing serum CRP. Furthermore, according to the SUCRA results, EHDS may be the best dosing strategy for vitamin D supplementation in terms of improving insulin resistance (HOMA-IR) and reducing inflammatory response (serum CRP).

Previous studies have shown that vitamin D supplementation was found to be safe. In the 2013 pilot study conducted by Coimbra’s research team [49], the aim was to evaluate the effects of prolonged, high daily doses of vitamin D on the clinical course of psoriasis and vitiligo. Their study involved nine patients with psoriasis and sixteen patients with vitiligo who were administered 35,000 IU of vitamin D3 daily for six months in conjunction with a low-calcium diet (avoiding dairy products and foods enriched with calcium such as oats, rice, or soya "milk") and consistent hydration (at least 2.5 L daily). There were no changes observed in serum levels of creatinine or calcium (total and ionized), while urinary calcium excretion levels did increase within the normal range [49]. One study performed by Ulrich Amon et al. has shown high doses of orally administered vitamin D3 up to 1000 IU/kg weight are safe from a perspective of calcium metabolism and renal function in over 300 patients with various autoimmune diseases, when strict recommendations for diet and fluid intake are followed, up to a treatment period of 3.5 years [50].

Studies [51, 52] reveal that low vitamin D status is inversely associated with insulin resistance in children and adolescents. Therefore, vitamin D supplementation is recommended for preventing insulin resistance in this population because lower serum concentrations of 25-(OH)-D are common in subjects with overweight/obesity [53]. In the current network meta-analysis, we find that EHDS (> 4000 IU/day) significantly increases serum 25-(OH)-D levels and has the highest probability of ranking best. As a result, it is reasonable to speculate that vitamin D supplementation benefits improving insulin resistance. Our network meta-analysis does find that EHDS (> 4000 IU/day) significantly decreases HOMA-IR, suggesting an improvement in insulin resistance. In addition, a previous meta-analysis [14] also suggests that vitamin D supplementation, especially extremely high doses of more than 4000 IU/day, benefits improving insulin resistance in children and adolescents.

It is worth noting that there has yet to be a consensus on dose levels of vitamin D supplementation in children and adolescents [54]. The previous meta-analysis performs subgroup analyses to evaluate the efficacy of different dosage strategies of vitamin D supplementation in children and adolescents with overweight/obesity, but the direct comparison between different dosage strategies is lacking, making it impossible to determine the relative efficacy between different dosage strategies. In the current network meta-analysis, we simultaneously compared multiple dosage strategies by combining direct and indirect evidence. We found that EHDS (> 4000 IU/day) has the highest probability of being the best dosage strategy in increasing serum 25-(OH)-D levels and improving insulin resistance.

The possible mechanisms of vitamin D supplementation in decreasing insulin resistance have yet to be clarified [34]. However, studies have found that inflammatory cytokines such as CRP are associated with obesity and insulin resistance and are involved in modulating the effects of vitamin D on insulin resistance [34, 55]. The current network meta-analysis found that EHDS (> 4000 IU/day) significantly decreased the concentration of serum CRP and ranked better than LDS (< 1000 IU/day) and MDS (1000–2000 IU/day). The previous meta-analysis [14] consistently suggested that supplementing vitamin D with a dose of greater than 4000 IU/day could reduce CRP. Research on vitamin D supplementation in adults with overweight/obesity has shown a reduction in bioavailability. Lang et al. also discovered that the level of obesity had a significant impact on the pharmacokinetics of 25(OH)D. The average 25(OH)D concentrations declined with increasing BMI z-scores, indicating that children with severe obesity (BMI z-scores > 3) may require higher doses to attain 25(OH)D concentrations ≥ 40 ng/mL. It is suggested that for patients with BMI z-scores > 3, an additional 1–2 loading doses per week may be deemed appropriate if the prompt attainment of target levels of 25(OH)D ≥ 40 ng/mL is of critical importance [56]. Overall, based on these findings, we can reasonably believe that EHDS (> 4000 IU/day) should be preferentially recommended to prevent or improve insulin resistance because this dosage strategy benefits increasing the concentration of serum 25-(OH)-D and reducing the level of CRP in children and adolescents with overweight/obesity. A total of seven of the original studies in our meta-analysis reported parathyroid hormone (PTH) outcomes, namely Samaranayake et al., 2020 (2500 IU per week, 50,000 IU per week and control), Brar et al., 2018 (300,000 once and control), Shah et al., 2015 (150,000 IU twice and control), Nader et al. 2014 (control and 2000 IU per day), Rajakumar et al., 2020 (600 IU per day, 1000 IU per day and 2000 IU per day), Varshney et al., 2019 (12,000 IU per month and 120,000 IU once), Asgharu et al., 2021 (600 IU per day, 1000 IU per day, and 1000 IU and 2000 IU per day). The results showed that there were no significant changes in PTH concentrations despite differences in vitamin D supplementation doses. Another study gave different results, Marwaha et al. found a significant decrease in PTH in vitamin D-deficient children after 6 months of supplementation with 600, 1000, and 2000 IU per day [57]. Further observation of the study population revealed that the subjects had vitamin D deficiency or high baseline serum PTH levels. One of the included studies (Asgharu et al., 2021) discussed the reasons, concluding that "the physiological process of PTH secretion in puberty is complex and not always easy to explain; it may be relatively independent of vitamin D dosage". We agree with the view that, first, the effect of calcium supplementation on bone metabolism is a complex physiological process, and second, the effect on PTH is one of the negative feedbacks in the metabolic process; therefore, the effect of calcium supplementation on PTH is not direct. In addition, the patient's age, pre-existing PTH level and overall metabolic level are all possible factors in the change of PTH.

Our network meta-analyses have four major methodological advantages: (i) we included only RCTs for data analysis, thereby greatly enhancing the strength of evidence; (ii) we combined both direct and indirect evidence improving the statistical power to estimate relative efficacy; (iii) we used indirect comparisons to estimate comparative efficacy of two dosage strategies that had never been directly compared; and (iv) we used SUCRA to calculate ranked probability of all the dosage strategies of vitamin D supplements, benefiting from identifying the optimal dosage strategy. The current network meta-analysis has some limitations. First, although only RCTs were included in our network meta-analysis, most of the included studies were judged as having “some concerns” or “high risk of bias”, thereby inevitably compromising the reliability of pooled results, suggesting the need to design more RCTs with high quality. Second, our network meta-analysis included 15 RCTs in the final analysis; however, the sample sizes of most studies were extremely inadequate, so the reliability of pooled results was inevitably compromised by inadequate sample size. Third, our network meta-analysis searched four recommended databases for study retrieval; however, only studies published in English were included, so publication bias was not avoidable. Fourth, loop inconsistency was available for the network meta-analysis of 25-(OH)-D and CRP. Therefore, the robustness of pooled results may be negatively influenced by inconsistency. Fifth, subgroup analysis cannot be designed due to limited studies, although variations in formula and route of administration. Therefore, more future studies are required to explore the influence of these variations on the efficacy of different dosage strategies in children and adolescents with overweight/obesity. Sixth, previous studies [58, 59] revealed that the duration of vitamin D supplementation is an important factor for evaluating the efficacy of vitamin D; however, we do not perform additional analysis based on the different durations of supplementation due to limited studies, which may introduce confounding for pooled results. It is also noted that although some studies have shown the safety of vitamin D in the healthy pediatric group, hemodialysis patients, and patients with type 2 diabetes [15], the safety of vitamin D especially high-dose supplementation in children and adolescents with overweight/obesity should be further investigated because the duration of obesity treatment is likely to for many years. Seventh, the current network meta-analysis does not conduct a safety assessment for using different dosage strategies because very few studies reported adverse events. Finally, this network meta-analysis did not register formal protocol, which inevitably compromised the transparency of our study although we conducted and reported pooled results in strict accordance with the PRISMA-NMA checklist.

## Conclusions

Based on the available data, our network meta-analysis concludes that EHDS may be the optimal dosing strategy for vitamin D supplementation to reduce the inflammatory response (CRP) and improve insulin resistance in children and adolescents with overweight/obesity. However, future studies are required to validate our findings. However, our findings in the current network meta-analysis need more validation in future studies with larger sample sizes and higher methodological quality.

## Data availability statement

All data generated or analyzed during this study are included in this published article/as supplementary information files.

### Supplementary Information

Below is the link to the electronic supplementary material.

Supplementary file1 (DOCX 2744 KB)

## Data Availability

Not applicable.
