# Supplementary material for: Optimal vitamin D supplement dosage for improving insulin resistance in children and adolescents with overweight/obesity: a systematic review and network meta-analysis
Source: Eur J Nutr. 2023 Dec 30;63(3):763–75. doi: 10.1007/s00394-023-03301-x (PMC10948536; doi:10.1007/s00394-023-03301-x)
Supplement: Supplementary file 1 — Supplementary file1 (DOCX 2744 KB) [file 394_2023_3301_MOESM1_ESM.docx]

**
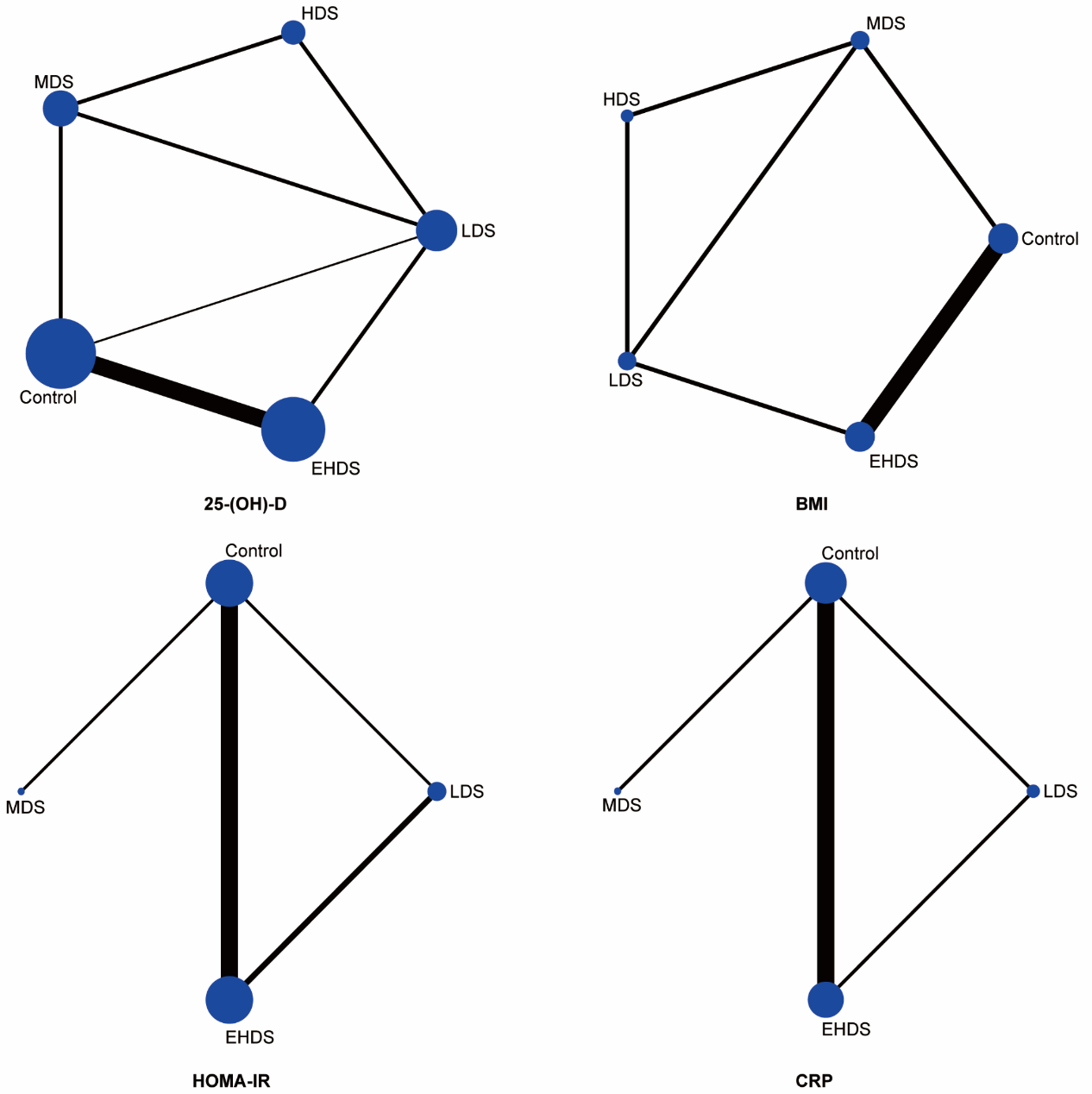
Supplementary Figure 1.** Evidence plots of 25-(OH)-D, BMI, HOMA-IR, and CRP. BMI, body mass index, HOMA-IR, homeostasis model assessment-insulin resistance; CRP, C-reactive protein.


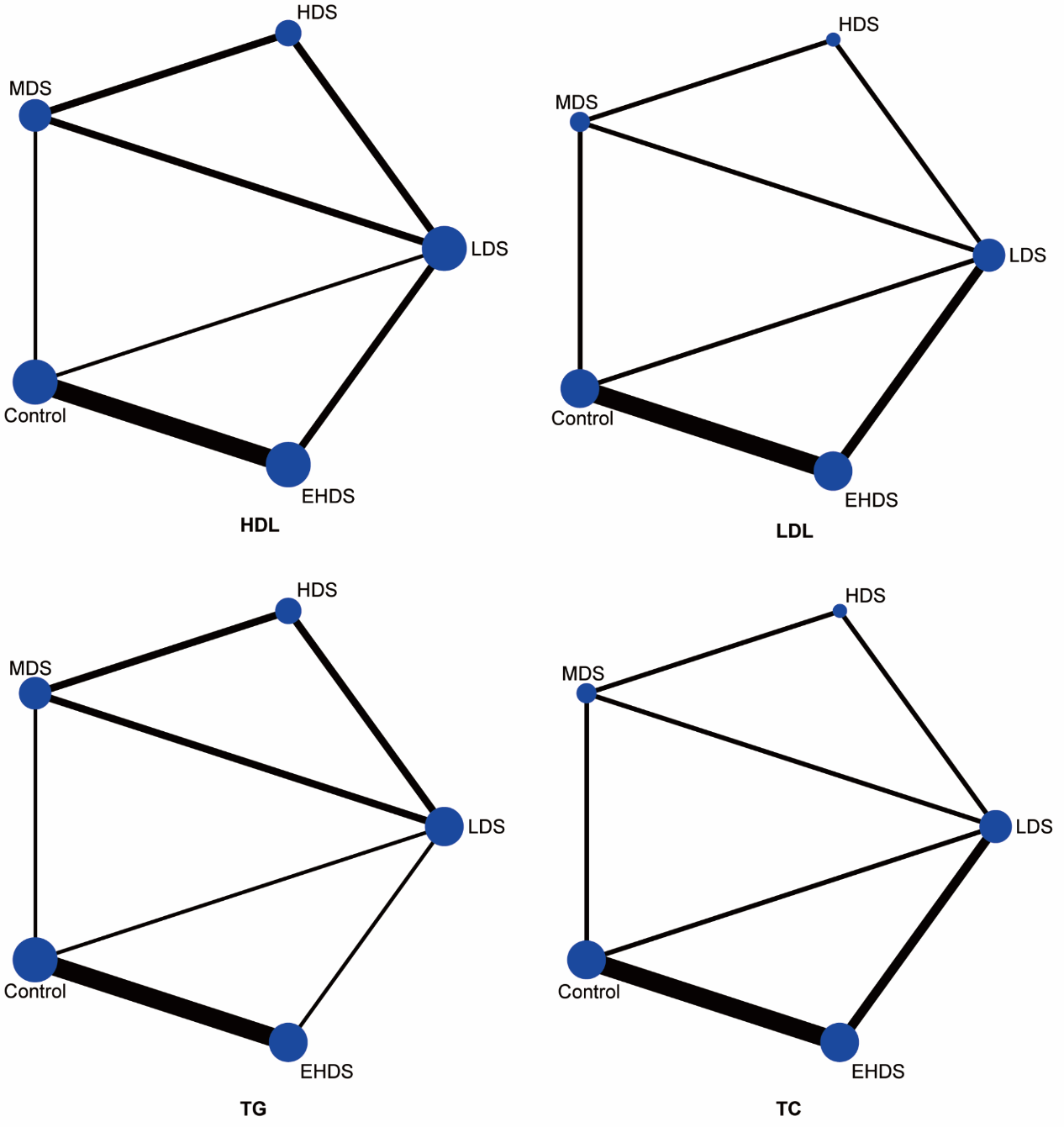


**Supplementary Figure 2.** Evidence plots of cardiometabolic factors, including HDL, LDL, TG, and TC. HDL, high-density lipoprotein; LDL, low-density lipoprotein; TG, triglyceride; TC, total cholesterol.


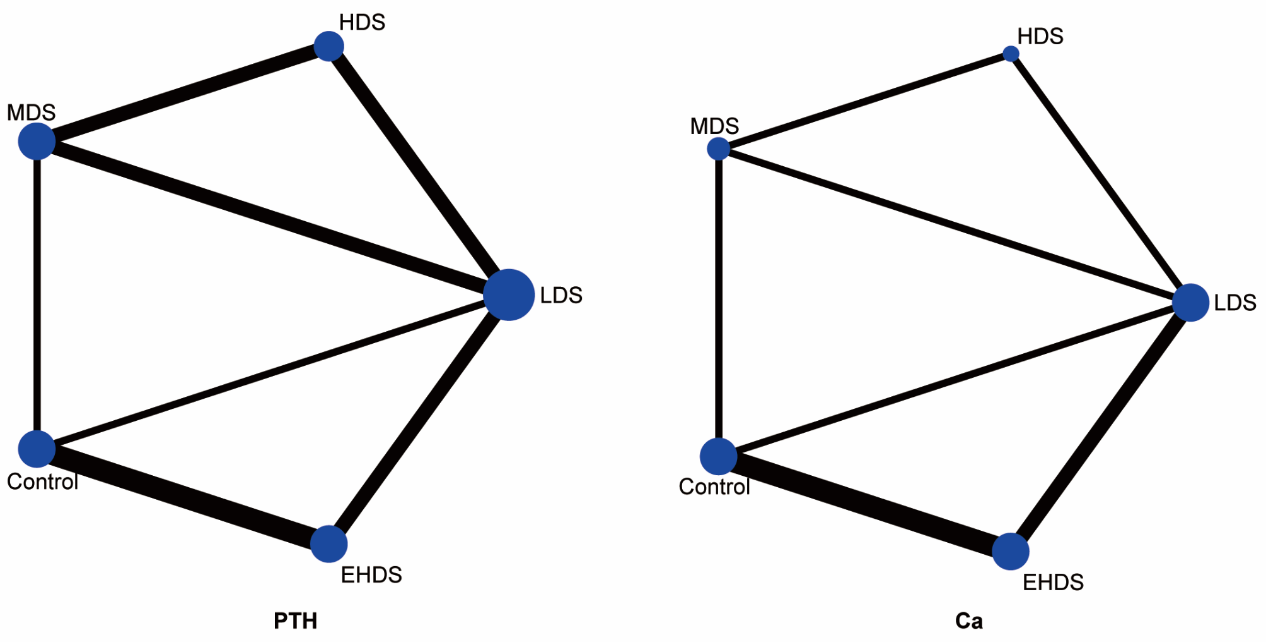


**Supplementary Figure 3.** Evidence plots of bone metabolism-associated markers, including PTH and Ca. PTH, parathyroid hormone; Ca, calcium.


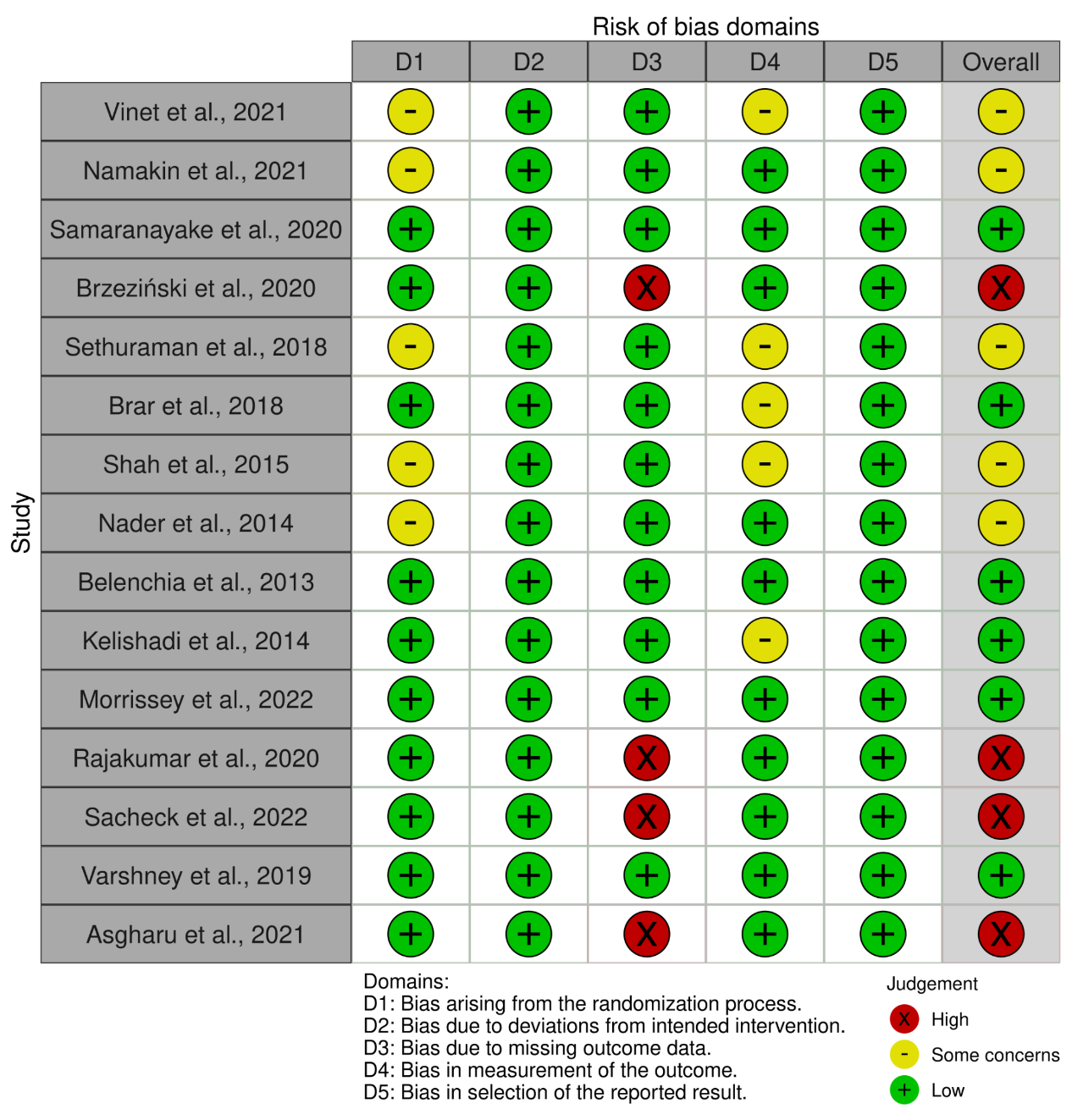


**Supplementary Figure 4.** Risk of bias assessment of included studies.


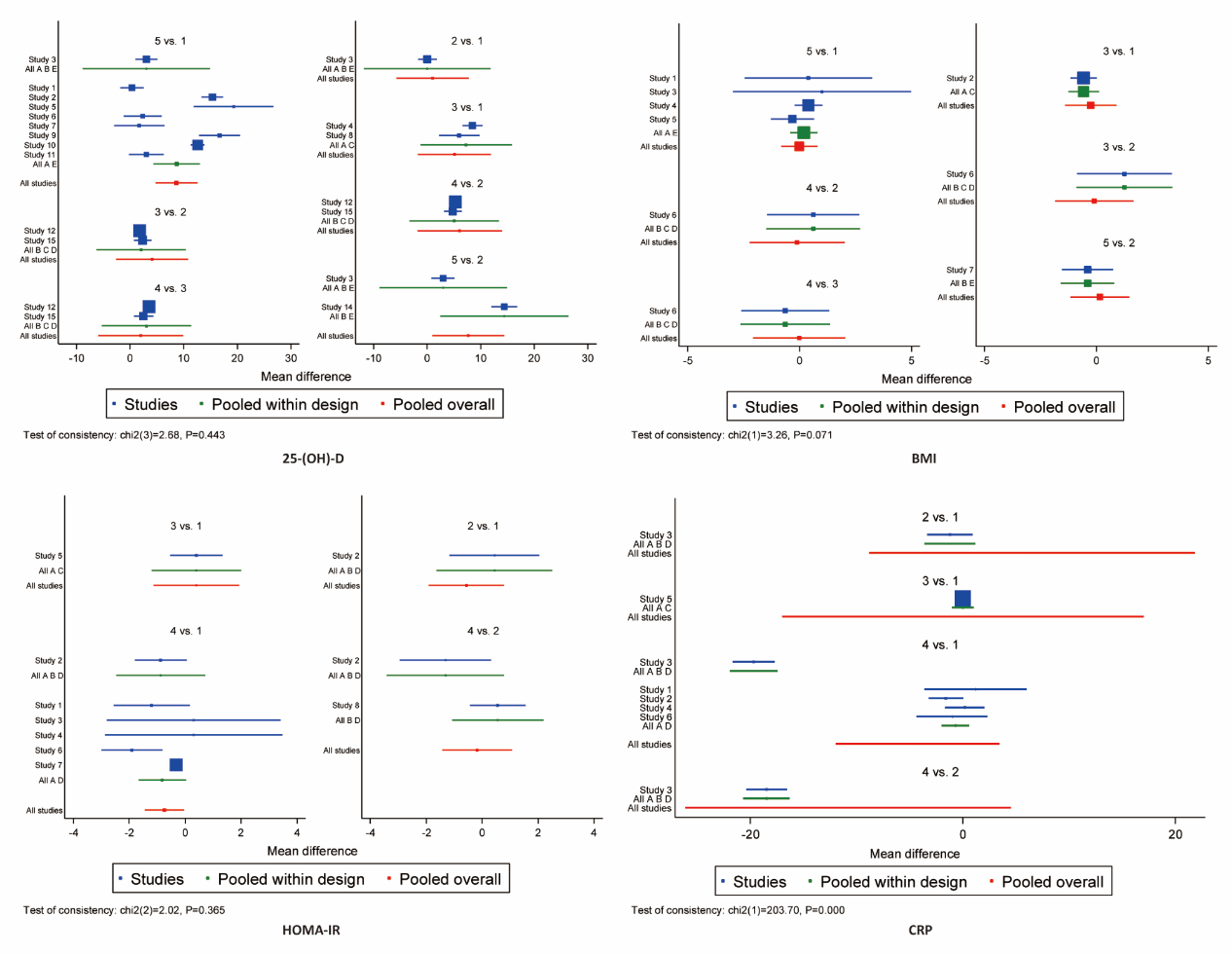


**Supplementary Figure 5.** Global consistency model test for 25-(OH)-D, BMI, HOMA-IR, and CRP. BMI, body mass index, HOMA-IR, homeostasis model assessment-insulin resistance; CRP, C-reactive protein.


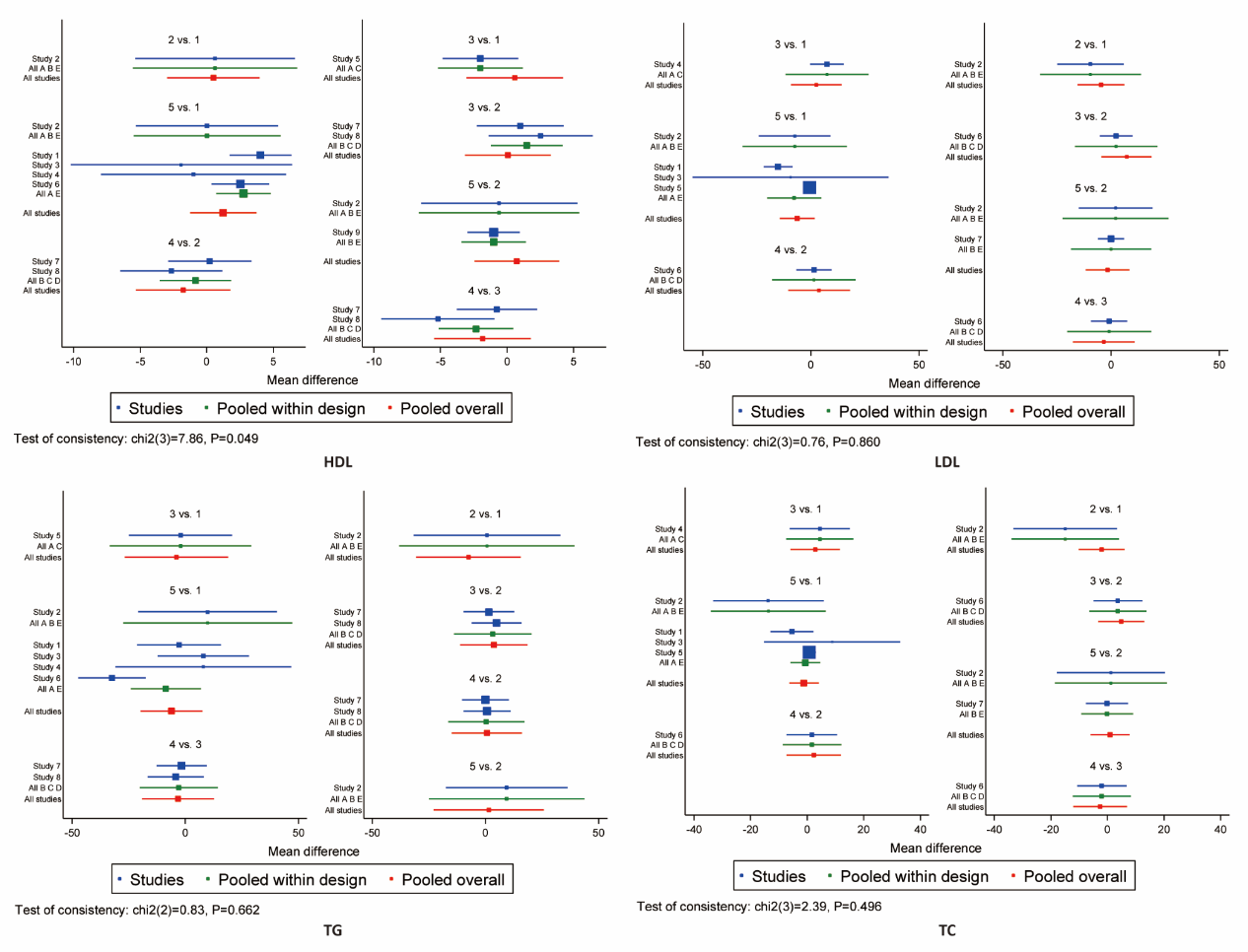


**Supplementary Figure 6.** Global consistency model test for cardiometabolic factors, including HDL, LDL, TG, and TC. HDL, high-density lipoprotein; LDL, low-density lipoprotein; TG, triglyceride; TC, total cholesterol.


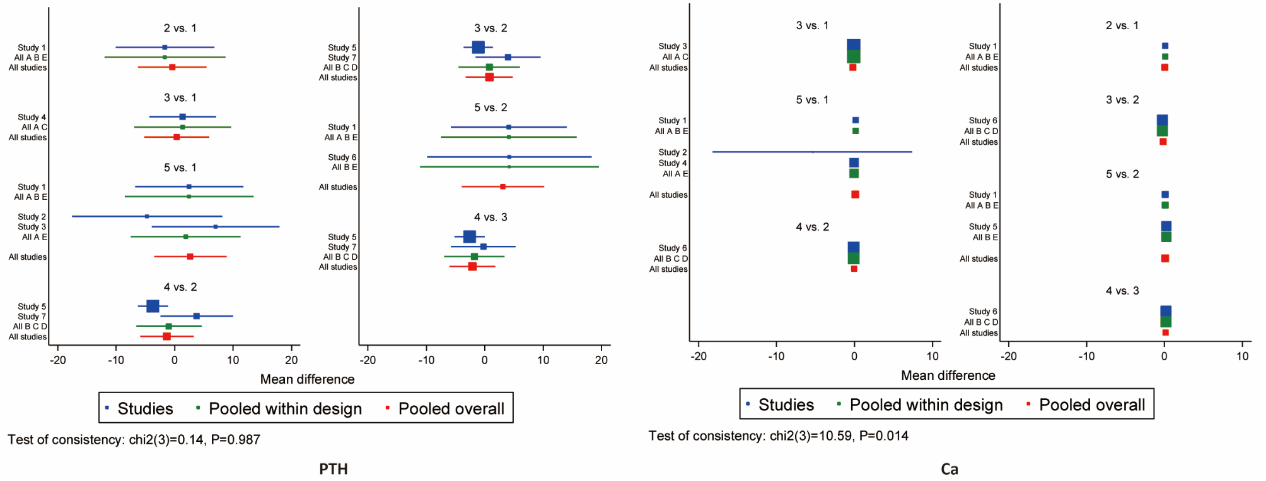


**Supplementary Figure 7.** Global consistency model test for bone metabolism-associated markers, including PTH and Ca. PTH, parathyroid hormone; Ca, calcium.


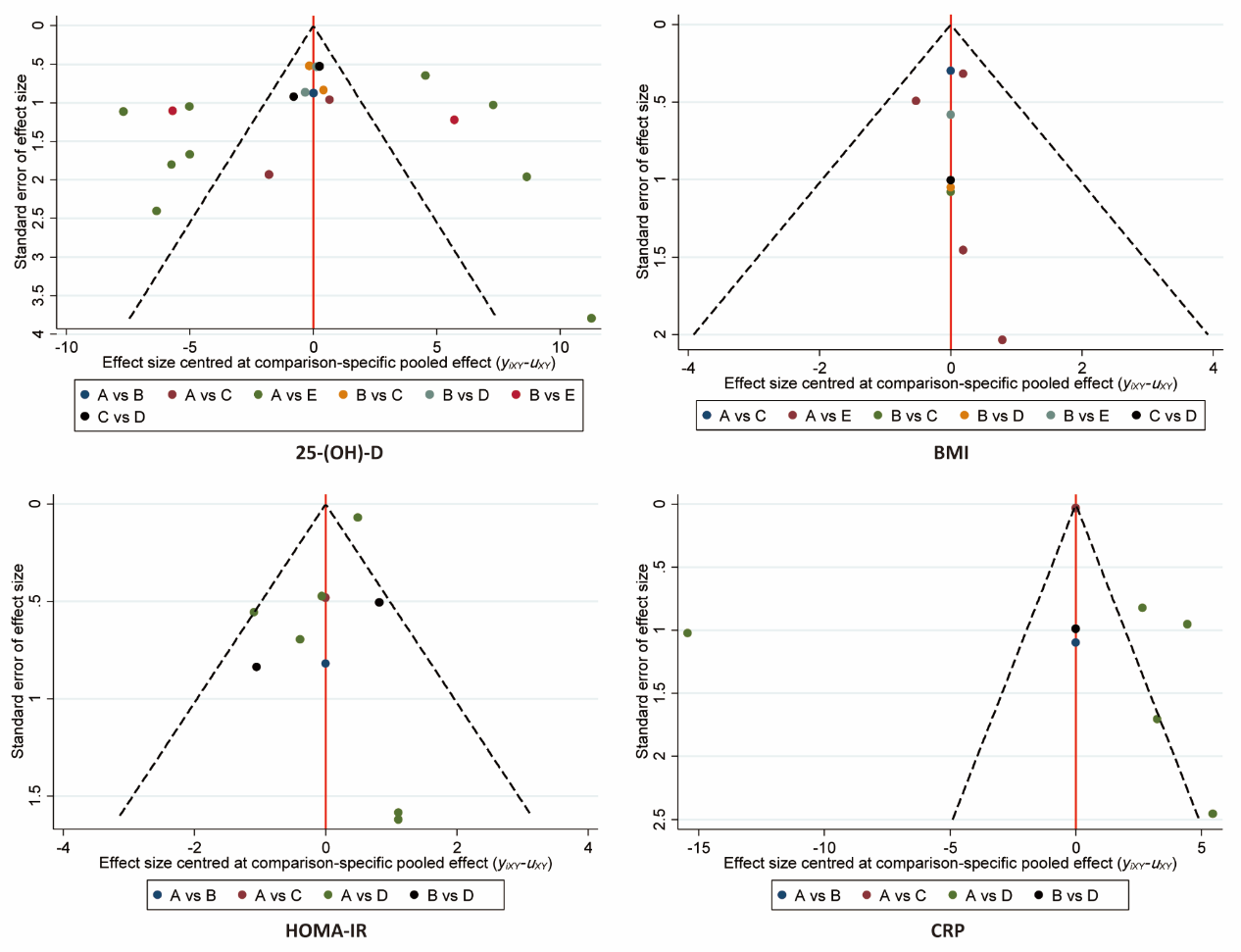


**Supplementary Figure 8.** Comparison-adjusted funnel plots for 25-(OH)-D, BMI, HOMA-IR, and CRP. BMI, body mass index, HOMA-IR, homeostasis model assessment-insulin resistance; CRP, C-reactive protein.


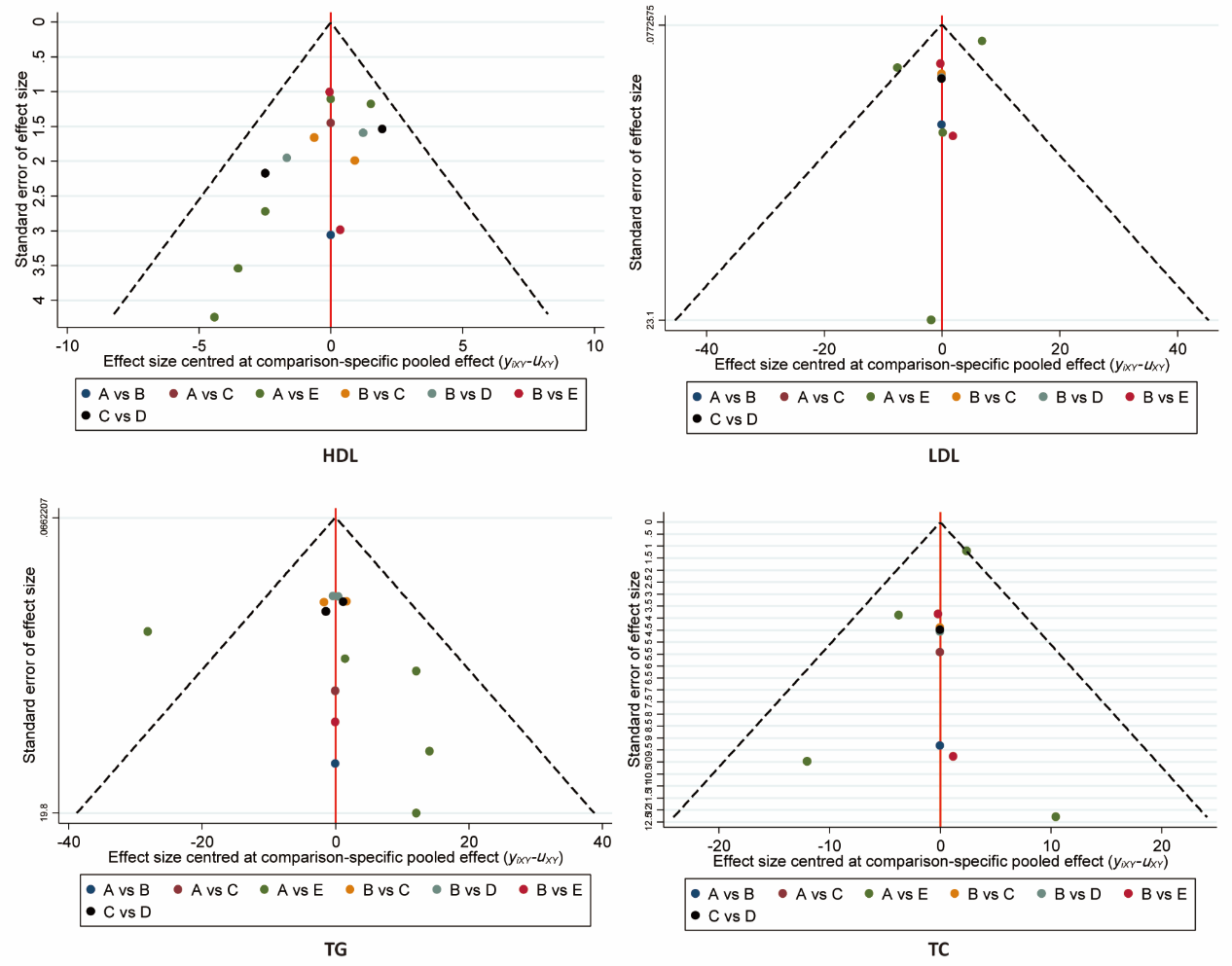


**Supplementary Figure 9.** Comparison-adjusted funnel plots for cardiometabolic factors, including HDL, LDL, TG, and TC. HDL, high-density lipoprotein; LDL, low-density lipoprotein; TG, triglyceride; TC, total cholesterol.


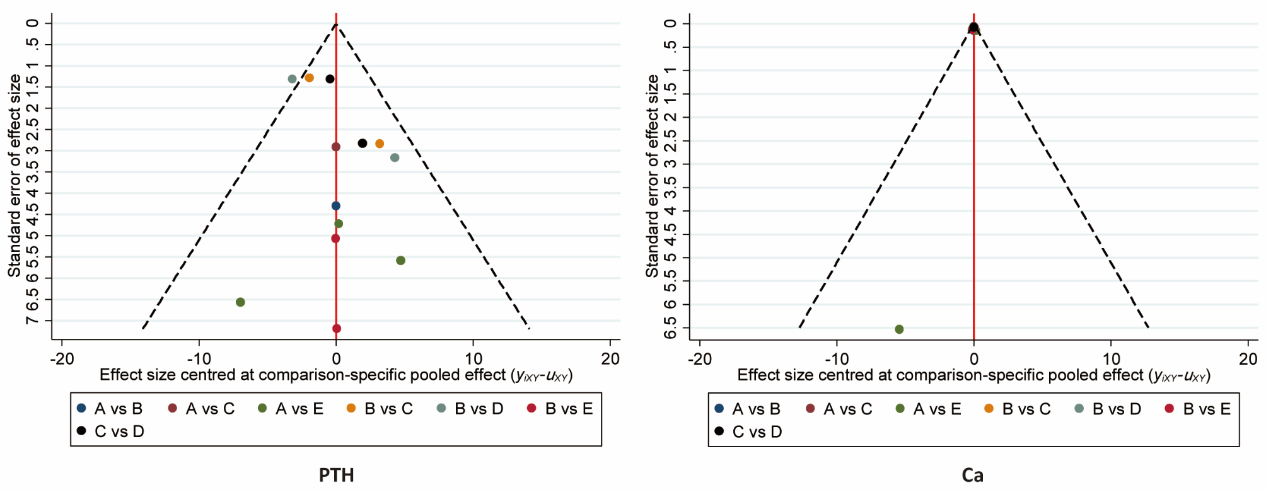


**Supplementary Figure 10.** Comparison-adjusted funnel plots for bone metabolism-associated markers, including PTH and Ca. PTH, parathyroid hormone; Ca, calcium.

**Supplementary Table 1.** Detailed search strategies of all target databases.

PubMed

| #No. | Search Details | Results |
| --- | --- | --- |
| #1 | "Vitamin D"[MeSH Terms] OR "Cholecalciferol"[MeSH Terms] OR "Ergocalciferols"[MeSH Terms] OR "25-Hydroxyvitamin D 2"[MeSH Terms] OR "Dihydrotachysterol"[MeSH Terms] OR "Hydroxycholecalciferols"[MeSH Terms] OR "Calcitriol"[MeSH Terms] | 67,101 |
| #2 | ("vitam"[All Fields] AND "D"[Title/Abstract]) OR "Cholecalciferol"[Title/Abstract] OR "Cholecalciferols"[Title/Abstract] OR "Calciol"[Title/Abstract] OR "Ergocalciferol"[Title/Abstract] OR "Calciferol"[Title/Abstract] OR "Calciferols"[Title/Abstract] OR "Hydroxyvitamin"[Title/Abstract] OR "Hydroxyergocalciferol"[Title/Abstract] OR "Ercalcidiol"[Title/Abstract] OR "Hydroxycalciferol"[Title/Abstract] OR "Tachystin"[Title/Abstract] OR "Dihydrotachysterin"[Title/Abstract] OR "Calcamine"[Title/Abstract] OR "Calcidiol"[Title/Abstract] OR "Dedrogyl"[Title/Abstract] OR "Hidroferol"[Title/Abstract] OR "Calderol"[Title/Abstract] | 20,822 |
| #3 | #1 or #2 | 71,642 |
| #4 | "Overweight"[MeSH Terms] OR "Obesity"[MeSH Terms] | 263,030 |
| #5 | "obesity"[Title/Abstract] OR "overweight"[Title/Abstract] OR "obese"[Title/Abstract] OR "adipose tissue hyperplasia"[Title/Abstract] OR "adipositas"[Title/Abstract] OR "adiposity"[Title/Abstract] OR "body weight excess"[Title/Abstract] OR "corpulency"[Title/Abstract] OR "fat overload syndrome"[Title/Abstract] OR "obesitas"[Title/Abstract] | 399,087 |
| #6 | #4 or #5 | 447,190 |
| #7 | "Child"[MeSH Terms] | 2,113,384 |
| #8 | "child"[Title/Abstract] OR "children"[Title/Abstract] OR "childhood"[Title/Abstract] | 1,595,907 |
| #9 | #7 or #8 | 2,624,253 |
| #10 | "Adolescent"[MeSH Terms] | 2,195,296 |
| #11 | "adolescent"[Title/Abstract] OR "adolescents"[Title/Abstract] OR "adolescence"[Title/Abstract] OR "teens"[Title/Abstract] OR "teen"[Title/Abstract] OR "teenagers"[Title/Abstract] OR "teenager"[Title/Abstract] OR "youth"[Title/Abstract] OR "youths"[Title/Abstract] OR "juvenile"[Title/Abstract] OR "juveniles"[Title/Abstract] OR "minors"[Title/Abstract] | 512,959 |
| #12 | #10 or #11 | 2,373,793 |
| #13 | #9 or #12 | 3,881,095 |
| #14 | "Randomized Controlled Trials as Topic"[MeSH Terms] OR "Randomized Controlled Trial"[Publication Type] OR "Random Allocation"[MeSH Terms] | 826,590 |
| #15 | "random allocation"[MeSH Terms] OR ("random"[All Fields] AND "allocation"[All Fields]) OR "random allocation"[All Fields] OR "randomization"[All Fields] OR "randomized"[All Fields] OR "random"[All Fields] OR "randomisation"[All Fields] OR "randomisations"[All Fields] OR "randomise"[All Fields] OR "randomised"[All Fields] OR "randomising"[All Fields] OR "randomizations"[All Fields] OR "randomize"[All Fields] OR "randomizes"[All Fields] OR "randomizing"[All Fields] OR "randomness"[All Fields] OR "randoms"[All Fields] | 1,417,823 |
| #16 | #14 or #15 | 1,419,375 |
| #17 | #3 and #6 and #13 and | 99 |
| #18 | #17 AND (humans[Filter]) | 95 |

EMBASE

| #No. | Search Details | Results |
| --- | --- | --- |
| #1 | 'vitamin d':ti,ab,kw OR cholecalciferol:ti,ab,kw OR cholecalciferols:ti,ab,kw OR calciol:ti,ab,kw OR ergocalciferol:ti,ab,kw OR calciferol:ti,ab,kw OR calciferols:ti,ab,kw OR hydroxyvitamin:ti,ab,kw OR hydroxyergocalciferol:ti,ab,kw OR ercalcidiol:ti,ab,kw OR hydroxycalciferol:ti,ab,kw OR tachystin:ti,ab,kw OR dihydrotachysterin:ti,ab,kw OR calcamine:ti,ab,kw OR calcidiol:ti,ab,kw OR dedrogyl:ti,ab,kw OR hidroferol:ti,ab,kw OR calderol:ti,ab,kw | 122,508 |
| #2 | 'vitamin d'/exp OR 'colecalciferol'/exp OR 'ergocalciferol'/exp OR '25 hydroxyvitamin d'/exp OR 'dihydrotachysterol'/exp OR 'hydroxycolecalciferol'/exp OR 'calcitriol'/exp | 172,790 |
| #3 | #1 OR #2 | 191,559 |
| #4 | obesity:ti,ab,kw OR overweight:ti,ab,kw OR obese:ti,ab,kw OR 'adipose tissue hyperplasia':ti,ab,kw OR adipositas:ti,ab,kw OR adiposity:ti,ab,kw OR 'body weight excess':ti,ab,kw OR corpulency:ti,ab,kw OR 'fat overload syndrome':ti,ab,kw OR obesitas:ti,ab,kw OR calcidiol:ti,ab,kw | 593,468 |
| #5 | 'obesity'/exp | 631,402 |
| #6 | #4 OR #5 | 760,022 |
| #7 | child:ti,ab,kw OR children:ti,ab,kw OR childhood:ti,ab,kw | 2,084,448 |
| #8 | 'child'/exp | 3,264,484 |
| #9 | #7 OR #8 | 3,813,150 |
| #10 | adolescent:ti,ab,kw OR adolescents:ti,ab,kw OR adolescence:ti,ab,kw OR teens:ti,ab,kw OR teen:ti,ab,kw OR teenagers:ti,ab,kw OR teenager:ti,ab,kw OR youth:ti,ab,kw OR youths:ti,ab,kw OR juvenile:ti,ab,kw OR juveniles:ti,ab,kw OR minors:ti,ab,kw | 650,234 |
| #11 | 'adolescent'/exp | 1,867,249 |
| #12 | #10 OR #11 | 2,107,736 |
| #13 | #9 OR #12 | 4,789,430 |
| #14 | random | 420,264 |
| #15 | 'randomized controlled trial'/exp OR 'randomized controlled trial (topic)'/exp OR 'randomization'/exp | 1,049,863 |
| #16 | #14 OR #15 | 1,417,886 |
| #17 | #3 AND #6 AND #13 AND #16 | 254 |
| #18 | #17 AND [embase]/lim AND 'human'/de | 230 |

Cochrane library

| #No. | Search Details | Results |
| --- | --- | --- |
| #1 | (vitam D):ti,ab,kw OR (Cholecalciferol):ti,ab,kw OR (Cholecalciferols):ti,ab,kw OR (Calciol):ti,ab,kw OR (Ergocalciferol):ti,ab,kw | 3,646 |
| #2 | (Calciferol):ti,ab,kw OR (Calciferols):ti,ab,kw OR (Hydroxyvitamin):ti,ab,kw OR (Hydroxyergocalciferol):ti,ab,kw OR (Ercalcidiol):ti,ab,kw | 3,351 |
| #3 | (Hydroxycalciferol):ti,ab,kw OR (Tachystin):ti,ab,kw OR (Dihydrotachysterin):ti,ab,kw OR (Calcamine):ti,ab,kw OR (Calcidiol):ti,ab,kw | 100 |
| #4 | (Dedrogyl):ti,ab,kw OR (Hidroferol):ti,ab,kw OR (Calderol):ti,ab,kw | 18 |
| #5 | #1 or #2 or #3 or #4 | 5,881 |
| #6 | MeSH descriptor: [Vitamin D] explode all trees | 6,238 |
| #7 | MeSH descriptor: [Cholecalciferol] explode all trees | 3,512 |
| #8 | MeSH descriptor: [Ergocalciferols] explode all trees | 1,611 |
| #9 | MeSH descriptor: [25-Hydroxyvitamin D 2] explode all trees | 78 |
| #10 | MeSH descriptor: [Dihydrotachysterol] explode all trees | 15 |
| #11 | MeSH descriptor: [Hydroxycholecalciferols] explode all trees | 1,588 |
| #12 | MeSH descriptor: [Calcitriol] explode all trees | 971 |
| #13 | #5 or #6 or #7 or #8 or #9 or #10 or #11 or #12 | 8,810 |
| #14 | (obesity):ti,ab,kw OR (overweight):ti,ab,kw OR (obese):ti,ab,kw OR (adipose tissue hyperplasia):ti,ab,kw OR (adipositas):ti,ab,kw | 53,849 |
| #15 | (adiposity):ti,ab,kw OR (body weight excess):ti,ab,kw OR (corpulency):ti,ab,kw OR (fat overload syndrome):ti,ab,kw OR (obesitas):ti,ab,kw | 4,745 |
| #16 | #14 or #15 | 55,239 |
| #17 | MeSH descriptor: [Overweight] explode all trees | 19,364 |
| #18 | MeSH descriptor: [Obesity] explode all trees | 16,205 |
| #19 | # 16 or #17 or #18 | 268,007 |
| #20 | (child):ti,ab,kw OR (children):ti,ab,kw OR (childhood):ti,ab,kw OR (adolescent):ti,ab,kw OR (adolescents):ti,ab,kw | 268,698 |
| #21 | (adolescence):ti,ab,kw OR (teens):ti,ab,kw OR (teen):ti,ab,kw OR (teenagers):ti,ab,kw OR (teenager):ti,ab,kw | 8,830 |
| #22 | (youth):ti,ab,kw OR (youths):ti,ab,kw OR (juvenile):ti,ab,kw OR (juveniles):ti,ab,kw OR (minors):ti,ab,kw | 11,764 |
| #23 | #20 or #21 or #22 | 273,629 |
| #24 | MeSH descriptor: [Child] explode all trees | 62,328 |
| #25 | MeSH descriptor: [Adolescent] explode all trees | 110,852 |
| #26 | #23 or #24 or #25 | 273,629 |
| #27 | #13 and #19 and #26 in Trials | 335 |

Web of Science

| #No. | Search Query | Results |
| --- | --- | --- |
| #1 | Vitamin D (Topic) OR Cholecalciferol (Topic) OR Cholecalciferols (Topic) OR Calciol (Topic) OR Ergocalciferol (Topic) OR Calciferol (Topic) OR Calciferols (Topic) OR Hydroxyvitamin (Topic) OR Hydroxyergocalcifero (Topic) OR Ercalcidio (Topic) OR Hydroxycalciferol (Topic) OR Tachystin (Topic) OR Dihydrotachysterin (Topic) OR Calcamine (Topic) OR Calcidiol (Topic) OR Dedrogyl (Topic) OR Hidroferol (Topic) OR Calderol (Topic) | 212,399 |
| #2 | obesity (Topic) OR overweight (Topic) OR obese (Topic) OR adipose tissue hyperplasia (Topic) OR adipositas (Topic) OR adiposity (Topic) OR body weight excess (Topic) OR corpulency (Topic) OR fat overload syndrome (Topic) OR obesitas (Topic) | 901,758 |
| #3 | child (Topic) OR children (Topic) OR childhood (Topic) OR adolescent (Topic) OR adolescents (Topic) OR adolescence (Topic) OR teens (Topic) OR teen (Topic) OR teenager (Topic) OR teenagers (Topic) OR youth (Topic) OR youths (Topic) OR juvenile (Topic) OR juveniles (Topic) OR minors (Topic) | 6,295,032 |
| #4 | Randomized Controlled Trials (Topic) OR Random Allocation (Topic) OR randomization (Topic) | 853,506 |
| #5 | #1 AND #2 AND #3 AND #4 | 322 |

**Supplementary Table 2.** Outcomes and corresponding data of included studies in this network meta-analysis.

| Study | Category of intervention | 25-(OH)-D, ng/mL | BMI, kg/m2 | HOMA-IR | CRP, mg/mL | HDL, mg/dL | LDL, mg/dL | TG, mg/dL | TC, mg/dL | PTH, pg/mL | Ca, mg/dL |
| --- | --- | --- | --- | --- | --- | --- | --- | --- | --- | --- | --- |
| Vinet et al., 2021 | Control | 11.04±6.30 | 32.70±3.85 | 3.40±1.26 | 4.40±5.78 | n.r. | n.r. | n.r. | n.r. | n.r. | n.r. |
|  | EHDS | 12.48±4.22 | 33.80±3.85 | 5.60±2.37 | 7.90±7.48 | n.r. | n.r. | n.r. | n.r. | n.r. | n.r. |
| Namakin et al., 2021 | Control | 11.29±5.49 | n.r. | n.r. | 2.11±2.13 | 39.50±7.07 | 93.96±23.75 | 148.16±65.46 | 164.56±29.25 | n.r. | n.r. |
|  | EHDS | 9.78±6.31 | n.r. | n.r. | 2.40±2.60 | 37.08±10.31 | 105.14±28.16 | 138.73±61.99 | 160.41±31.28 | n.r. | n.r. |
| Samaranayake et al., 2020 | Control | 15.47±2.78 | n.r. | 2.53±1.81 | 3.56±3.13 | 41.55±9.59 | 115.63±29.11 | 129.16±67.57 | 176.98±41.26 | 34.19±15.37 | 9.71±0.58 |
|  | LDS | 14.92±3.92 | n.r. | 3.45±2.12 | 4.41±3.53 | 38.48±7.28 | 138.11±33.63 | 108.36±46.76 | 198.27±39.39 | 36.41±21.47 | 9.64±0.46 |
|  | EHDS | 14.92±3.04 | n.r. | 2.60±1.79 | 3.83±4.36 | 41.53±10.22 | 123.11±32.24 | 103.53±57.79 | 183.94±35.09 | 37.24±18.13 | 9.72±0.48 |
| Brzeziński et al., 2020 | Control | 19.79±5.15 | 24.53±3.57 | n.r. | n.r. | n.r. | n.r. | n.r. | n.r. | n.r. | n.r. |
|  | MDS | 19.35±5.46 | 24.97±4.12 | n.r. | n.r. | n.r. | n.r. | n.r. | n.r. | n.r. | n.r. |
| Sethuraman et al., 2018 | Control | 12.40±3.80 | 38.20±7.70 | 4.80±3.48 | n.r. | 41.90±11.7 | 219.50±60.00 | 79.90±22.30 | 155.90±38.50 | n.r. | n.r. |
|  | EHDS | 12.10±3.80 | 35.40±5.10 | 5.10±3.11 | n.r. | 46.90±10.2 | 203.30±56.40 | 72.90±28.60 | 154.10±24.30 | n.r. | n.r. |
| Brar et al., 2018 | Control | 16.70±2.90 | 32.70±9.80 | 4.20±2.80 | n.r. | n.r. | n.r. | n.r. | n.r. | 50.90±15.80 | 9.40±0.40 |
|  | EHDS | 16.70±2.90 | 32.70±9.80 | 4.20±2.80 | n.r. | n.r. | n.r. | n.r. | n.r. | 50.90±15.80 | 9.40±0.40 |
| Shah et al., 2015 | Control | 24.20±2.40 | 31.00±4.92 | n.r. | 2.00±2.24 | 46.00±3.00 | n.r. | 105.00±14.00 | n.r. | 40.00±5.00 | n.r. |
|  | EHDS | 19.40±1.30 | 36.00±7.16 | n.r. | 4.10±3.13 | 42.00±3.00 | n.r. | 113.00±15.00 | n.r. | 46.00±4.00 | n.r. |
| Nader et al., 2014 | Control | 24.40±7.30 | 33.90±5.30 | 2.80±1.40 | 0.30±0.30 | 43.60±8.90 | 90.70±20.40 | 106.30±47.20 | 155.50±21.60 | 24.90±11.40 | 9.80±0.30 |
|  | MDS | 25.80±5.90 | 35.30±7.20 | 3.10±1.50 | 0.30±0.30 | 40.80±6.20 | 106.90±36.10 | 132.50±63.80 | 174.00±44.30 | 34.00±25.00 | 9.90±0.30 |
| Belenchia et al., 2013 | Control | 19.60±7.90 | 38.90±6.70 | 4.79±0.43 | n.r. | n.r. | n.r. | n.r. | n.r. | n.r. | 9.24±0.29 |
|  | EHDS | 19.20±6.30 | 39.50±5.10 | 5.12±0.40 | n.r. | n.r. | n.r. | n.r. | n.r. | n.r. | 9.30±0.34 |
| Kelishadi et al., 2014 | Control | 17.91±2.27 | 27.81±1.04 | 3.15±0.26 | n.r. | 48.72±4.12 | 95.68±2.87 | 143.15±23.26 | 164.18±5.18 | n.r. | n.r. |
|  | EHDS | 18.27±2.04 | 28.08±1.06 | 3.21±0.11 | n.r. | 47.06±4.01 | 97.01±4.19 | 141.21±24.15 | 161.50±3.21 | n.r. | n.r. |
| Morrissey et al., 2022 | Control | 11.04±6.30 | 32.70±3.85 | n.r. | 4.40±5.78 | n.r. | n.r. | n.r. | n.r. | n.r. | n.r. |
|  | EHDS | 12.48±4.22 | 33.80±3.85 | n.r. | 7.90±7.48 | n.r. | n.r. | n.r. | n.r. | n.r. | n.r. |
| Rajakumar et al., 2020 | LDS | 14.30±4.30 | 30.68±7.06 | n.r. | n.r. | 46.49±10.24 | 91.64±22.94 | 72.66±34.13 | 152.72±27.48 | 51.70±20.60 | 9.70±0.30 |
|  | MDS | 14.40±3.40 | 29.80±6.14 | n.r. | n.r. | 45.41±9.58 | 86.88±24.63 | 77.14±32.96 | 147.72±27.27 | 45.70±18.00 | 9.80±0.30 |
|  | HDS | 14.20±3.50 | 30.27±5.78 | n.r. | n.r. | 44.95±8.98 | 91.51±25.39 | 76.16±31.21 | 151.71±26.64 | 52.00±19.90 | 9.70±0.30 |
| Sacheck et al., 2022 | LDS | n.r. | n.r. | n.r. | n.r. | 47.91±11.31 | n.r. | 63.56±26.49 | n.r. | n.r. | n.r. |
|  | MDS | n.r. | n.r. | n.r. | n.r. | 42.06±13.48 | n.r. | 70.47±32.93 | n.r. | n.r. | n.r. |
|  | HDS | n.r. | n.r. | n.r. | n.r. | 47.62±14.71 | n.r. | 71.05±33.62 | n.r. | n.r. | n.r. |
| Varshney et al., 2019 | LDS | 9.01±5.59 | 30.36±4.27 | 4.60±2.48 | n.r. | 40.00±6.95 | 91.00±21.00 | n.r. | 155.00±23.65 | 65.45±60.52 | 9.57±0.52 |
|  | EHDS | 8.36±5.45 | 29.53±3.27 | 4.44±2.30 | n.r. | 40.00±4.09 | 95.00±23.50 | n.r. | 157.00±28.65 | 57.65±49.16 | 9.45±0.54 |
| Asghari et al., 2021 | LDS | 11.55±6.6 | n.r. | n.r. | n.r. | n.r. | n.r. | n.r. | n.r. | 42.06±28.41 | 9.90±0.63 |
|  | MDS | 11.69±7.82 | n.r. | n.r. | n.r. | n.r. | n.r. | n.r. | n.r. | 38.33±22.13 | 10.10±0.67 |
|  | HDS | 12.28±7.57 | n.r. | n.r. | n.r. | n.r. | n.r. | n.r. | n.r. | 37.27±29.44 | 9.90±0.65 |

LDS, low dose strategy; MDS, medium dose strategy; HDS, high dose strategy; EHDS, extremely high dose strategy; BMI, body mass index; HOMA-IR, homeostasis model assessment-insulin resistance; CRP, C-reactive protein; HDL, high-density lipoprotein; LDL, low-density lipoprotein; TG, triglyceride; TC, total cholesterol; PTH, parathyroid hormone; Ca, calcium; n.r., not reported.

**Supplementary Table 3.** Transitivity assessment based on four major characteristics

| Characteristics | Comparison | MD | *SD* | *P* |
| --- | --- | --- | --- | --- |
| Supplementation duration, months | Control *vs.* LDS | -2.273 | 0.745 | 0.059 |
|  | Control *vs.* MDS | -1.773 | 0.849 | 0.056 |
|  | Control *vs.* HDS | -2.273 | 0.971 | 0.057 |
|  | Control *vs.* EHDS | -0.623 | 1.050 | 0.560 |
|  | LDS *vs.* MDS | 0.500 | 0.632 | 0.452 |
|  | LDS *vs.* HDS | 0.000 | 0.000 | n.a. |
|  | LDS *vs.* EHDS | 1.650 | 1.384 | 0.255 |
|  | MDS *vs.* HDS | -0.500 | 0.843 | 0.575 |
|  | MDS *vs.* EHDS | 1.150 | 1.449 | 0.442 |
|  | HDS *vs.* EHDS | 1.650 | 1.809 | 0.381 |
| Mean age, years | Control *vs.* LDS | 2.153 | 1.145 | 0.087 |
|  | Control *vs.* MDS | 1.278 | 1.210 | 0.314 |
|  | Control *vs.* HDS | 1.928 | 1.543 | 0.243 |
|  | Control *vs.* EHDS | -0.410 | 0.866 | 0.643 |
|  | LDS *vs.* MDS | -0.875 | 1.702 | 0.625 |
|  | LDS *vs.* HDS | -0.225 | 2.173 | 0.922 |
|  | LDS *vs.* EHDS | -2.563 | 1.190 | 0.057 |
|  | MDS *vs.* HDS | 0.650 | 2.365 | 0.797 |
|  | MDS *vs.* EHDS | -1.688 | 1.261 | 0.211 |
|  | HDS *vs.* EHDS | -2.338 | 1.602 | 0.183 |
| BMI at baseline, kg/m2 | Control *vs.* LDS | 3.750 | 2.597 | 0.174 |
|  | Control *vs.* MDS | 3.450 | 2.862 | 0.251 |
|  | Control *vs.* HDS | 5.100 | 3.630 | 0.190 |
|  | Control *vs.* EHDS | -1.022 | 2.004 | 0.617 |
|  | LDS *vs.* MDS | -4.772 | 2.323 | 0.065 |
|  | LDS *vs.* HDS | -0.300 | 3.186 | 0.928 |
|  | LDS *vs.* EHDS | 1.350 | 3.371 | 0.709 |
|  | MDS *vs.* HDS | 1.650 | 4.546 | 0.735 |
|  | MDS *vs.* EHDS | -4.472 | 2.651 | 0.120 |
|  | HDS *vs.* EHDS | -6.122 | 3.221 | 0.090 |

LDS, low dose strategy; MDS, medium dose strategy; HDS, high dose strategy; EHDS, extremely high dose strategy; BMI, body mass index; MD, mean difference; SD, standard difference; n.a., not applicable.

**Supplementary Table 4.** Local consistency assessment of analyses for all outcomes

| Side | Direct effect | | Indirect effect | | Difference | |  |
| --- | --- | --- | --- | --- | --- | --- | --- |
|  | *coefficient* | *se* | *coefficient* | *se* | *coefficient* | *se* | *p* |
| **25-(OH)-D, ng/mL** | | | | | | | |
| A B | 0.030251 | 6.24469 | 1.47513 | 4.392299 | -1.44488 | 7.633043 | 0.850 |
| A C | 7.27522 | 4.355741 | 0.94317 | 5.994253 | 6.33205 | 7.409803 | 0.393 |
| A E | 7.993016 | 1.98415 | 19.62756 | 8.170087 | -11.6345 | 8.407904 | 0.166 |
| B C | 2.012882 | 4.252124 | 8.340603 | 6.075995 | -6.32772 | 7.41609 | 0.394 |
| B D | 5.016967 | 4.253457 | 17.62329 | 14.19809 | -12.6063 | 14.81899 | 0.395 |
| B E | 8.681777 | 4.416379 | 5.740649 | 6.08061 | 2.941128 | 7.520005 | 0.696 |
| C D | 3.004096 | 4.256214 | -9.66 | 14.19622 | 12.6641 | 14.8196 | 0.393 |
| **BMI, kg/m2** | | | | | | | |
| A C | -0.57 | 0.34835 | 1.841019 | 1.2889 | -2.41102 | 1.335145 | 0.071 |
| A E | 0.191059 | 0.309529 | -2.21978 | 1.298714 | 2.410842 | 1.335097 | 0.071 |
| B C | 1.25 | 1.093496 | -1.16105 | 0.766097 | 2.411052 | 1.335155 | 0.071 |
| B D | 0.61 | 1.066022 | -4.21206 | 2.383524 | 4.822064 | 2.670302 | 0.071 |
| B E | -0.4 | 0.608069 | 2.011007 | 1.188638 | -2.41101 | 1.335142 | 0.071 |
| C D | -0.64 | 1.019447 | 4.182038 | 2.443873 | -4.82204 | 2.67029 | 0.071 |
| **HOMA-IR** | | | | | | | |
| A B | 0.176954 | 1.046028 | -1.0886 | 0.912797 | 1.265553 | 1.337103 | 0.344 |
| A D | -0.80865 | 0.339695 | 2.865469 | 2.348912 | -3.67412 | 2.37615 | 0.122 |
| B D | -0.1787 | 0.720245 | -0.81696 | 1.921091 | 0.638263 | 1.949397 | 0.743 |
| **CRP, mg/mL** | | | | | | | |
| A B | -1.2 | 1.215393 | 36.77972 | 2.334998 | -37.9797 | 2.661043 | 0.000 |
| B D | -18.47 | 1.117096 | 19.50972 | 2.477912 | -37.9797 | 2.661043 | 0.000 |
| **HDL, mg/dL** | | | | | | | |
| A B | 0.517351 | 3.718505 | 0.431198 | 2.1314 | 0.086153 | 4.275112 | 0.984 |
| A C | -2 | 1.614931 | 4.67925 | 2.060264 | -6.67925 | 2.617764 | 0.011 |
| A E | 2.495275 | 1.061804 | -4.17783 | 2.410858 | 6.673107 | 2.602769 | 0.010 |
| B C | 1.487989 | 1.374802 | -5.19143 | 2.282896 | 6.679421 | 2.617723 | 0.011 |
| B D | -0.84163 | 1.350355 | -14.2001 | 5.002439 | 13.35849 | 5.235402 | 0.011 |
| B E | -0.94917 | 0.951213 | 6.164738 | 1.993872 | -7.1139 | 2.199299 | 0.001 |
| C D | -2.32964 | 1.409158 | 11.02886 | 5.151999 | -13.3585 | 5.23553 | 0.011 |
| **LDL, mg/dL** | | | | | | | |
| A B | -9.50119 | 10.39837 | -2.56331 | 6.956886 | -6.93788 | 12.49616 | 0.579 |
| A C | 7.5 | 7.476176 | -5.89701 | 9.838094 | 13.39701 | 12.35643 | 0.278 |
| A E | -7.44198 | 4.586656 | 2.957763 | 13.08784 | -10.3997 | 13.88072 | 0.454 |
| B C | 2.35 | 7.407008 | 15.74779 | 9.890794 | -13.3978 | 12.35684 | 0.278 |
| B D | 1.48 | 7.564668 | 28.27382 | 23.66324 | -26.7938 | 24.71252 | 0.278 |
| B E | 0.763161 | 6.167045 | -9.42711 | 11.28151 | 10.19027 | 12.90964 | 0.430 |
| C D | -0.87 | 7.622029 | -27.664 | 23.60831 | 26.79401 | 24.71285 | 0.278 |
| **TG, mg/dL** | | | | | | | |
| A B | -0.58416 | 19.70634 | -11.9741 | 16.01818 | 11.38995 | 25.87973 | 0.660 |
| A C | -2 | 16.09015 | -5.92095 | 18.90754 | 3.920947 | 24.82716 | 0.875 |
| A E | -5.881 | 7.472149 | 1.972529 | 48.77827 | -7.85353 | 49.65414 | 0.874 |
| B C | 3.202339 | 8.829778 | 7.127025 | 23.20723 | -3.92469 | 24.83035 | 0.874 |
| B D | 0.359996 | 8.711474 | 8.21352 | 48.92358 | -7.85352 | 49.65416 | 0.874 |
| B E | 9.774322 | 17.19068 | -7.5793 | 18.12883 | 17.35362 | 24.80926 | 0.484 |
| C D | -2.84236 | 8.934651 | -10.6843 | 48.79991 | 7.841893 | 49.65434 | 0.875 |
| **TC, mg/dL** | | | | | | | |
| A B | -14.7833 | 9.433589 | 0.560051 | 3.917197 | -15.3434 | 10.22217 | 0.133 |
| A C | 4.5 | 6.326659 | -0.04179 | 7.720188 | 4.541791 | 9.981379 | 0.649 |
| A E | -1.63658 | 3.129556 | -1.4093 | 9.47332 | -0.22728 | 9.93707 | 0.982 |
| B C | 3.7 | 5.47583 | 8.241925 | 8.345477 | -4.54193 | 9.981568 | 0.649 |
| B D | 1.71 | 5.613506 | 10.79343 | 19.19886 | -9.08343 | 19.96263 | 0.649 |
| B E | 0.283356 | 4.421242 | 3.074567 | 8.062468 | -2.79121 | 9.233545 | 0.762 |
| C D | -1.99 | 5.548413 | -11.0736 | 19.25562 | 9.083573 | 19.96271 | 0.649 |
| **PTH, pg/mL** | | | | | | | |
| A B | -1.60695 | 4.904171 | 0.158542 | 3.965516 | -1.76549 | 6.281495 | 0.779 |
| A C | 1.4 | 3.745958 | -1.23849 | 4.832974 | 2.638494 | 6.114723 | 0.666 |
| A E | 2.150389 | 3.430272 | 5.665327 | 8.174323 | -3.51494 | 8.766989 | 0.688 |
| B C | 0.570583 | 2.302346 | 3.209784 | 5.675973 | -2.6392 | 6.115338 | 0.666 |
| B D | -1.31242 | 2.502494 | 3.96579 | 11.95119 | -5.27821 | 12.22974 | 0.666 |
| B E | 4.206358 | 4.46539 | 1.066826 | 6.281203 | 3.139532 | 7.703111 | 0.684 |
| C D | -1.88294 | 2.210275 | -7.15992 | 12.10291 | 5.276988 | 12.22946 | 0.666 |
| **Ca, mg/dL** | | | | | | | |
| A B | 0.069446 | 0.217614 | -0.04017 | 0.185339 | 0.109621 | 0.285801 | 0.701 |
| A C | -0.1 | 0.123757 | -0.39504 | 0.190182 | 0.295041 | 0.226903 | 0.194 |
| A E | 0.009848 | 0.076721 | 0.450365 | 0.124191 | -0.44052 | 0.14574 | 0.003 |
| B C | -0.3 | 0.131577 | -0.00494 | 0.18486 | -0.29506 | 0.226904 | 0.193 |
| B D | -0.1 | 0.13058 | 0.490111 | 0.434295 | -0.59011 | 0.453794 | 0.193 |
| B E | 0.197991 | 0.068569 | -0.25127 | 0.131143 | 0.44926 | 0.147989 | 0.002 |
| C D | 0.2 | 0.131071 | -0.39008 | 0.433866 | 0.590081 | 0.453807 | 0.194 |

BMI, body mass index; HOMA-IR, homeostasis model assessment-insulin resistance; CRP, C-reactive protein; HDL, high-density lipoprotein; LDL, low-density lipoprotein; TG, triglyceride; TC, ; total cholesterol; PTH, parathyroid hormone; Ca, calcium; se, standard error.

**Supplementary Table 5.** Loop inconsistency assessment of analyses for all outcomes

| Loop | *IF* | *seIF* | 95%CI | *p* | *tau2* |
| --- | --- | --- | --- | --- | --- |
| **25-(OH)-D, ng/mL** | | | | | |
| Control-LDS-MDS | 6.044 | 1.303 | (3.49, 8.60) | 0.000 | 0.000 |
| Control-LDS-EHDS | 0.009 | 9.651 | (0.00, 18.92) | 0.999 | 48.291 |
| **BMI, kg/m2** | | | | | |
| Control-LDS-MDS-EHDS | 2.429 | 1.287 | (0.00, 4.95) | 0.059 | 0.000 |
| **HOMA-IR** | | | | | |
| Control-LDS-EHDS | 1.078 | 1.423 | (0.00, 3.87) | 0.449 | 0.603 |
| **CRP, mg/mL** | | | | | |
| Control-LDS-EHDS | 18.941 | 1.580 | (15.84, 22.04) | 0.000 | 0.000 |
| **HDL, mg/dL** | | | | | |
| Control-LDS-MDS | 4.236 | 3.619 | (0.00, 11.33) | 0.242 | 0.000 |
| Control-LDS-EHDS | 3.196 | 3.297 | (0.00, 9.66) | 0.332 | 0.000 |
| **LDL, mg/dL** | | | | | |
| Control-LDS-MDS | 14.690 | 9.603 | (0.00, 33.51) | 0.126 | 0.000 |
| Control-LDS-EHDS | 1.198 | 14.929 | (0.00, 30.46) | 0.936 | 72.058 |
| **TG, mg/dL** | | | | | |
| Control-LDS-MDS | 16.807 | 36.012 | (0.00, 87.39) | 0.641 | 357.010 |
| Control-LDS-EHDS | 5.823 | 20.581 | (0.00, 46.16) | 0.777 | 0.000 |
| **TC, mg/dL** | | | | | |
| Control-LDS-MDS | 15.730 | 11.644 | (0.00, 38.55) | 0.177 | 0.000 |
| Control-LDS-EHDS | 15.147 | 10.039 | (0.00, 34.82) | 0.131 | 0.000 |
| **PTH, pg/mL** | | | | | |
| Control-LDS-MDS | 3.275 | 5.138 | (0.00, 13.70) | 0.538 | 0.000 |
| Control-LDS-EHDS | 0.429 | 7.327 | (0.00, 14.79) | 0.953 | 0.000 |
| **Ca, mg/dL** | | | | | |
| Control-LDS-MDS | 0.319 | 0.180 | (0.00, 0.67) | 0.076 | 0.000 |
| Control-LDS-EHDS | 0.130 | 0.167 | (0.00, 0.46) | 0.436 | 0.000 |

BMI, body mass index; HOMA-IR, homeostasis model assessment-insulin resistance; CRP, C-reactive protein; HDL, high-density lipoprotein; LDL, low-density lipoprotein; TG, triglyceride; TC, ; total cholesterol; PTH, parathyroid hormone; Ca, calcium; IF, inconsistency factor; se, standard error; CI, confidence interval; LDS, low dose strategy; MDS, medium dose strategy; EHDS, extremely high dose.
